# The impact of musical training on reading ability in children: the mediating role of working memory

**DOI:** 10.3389/fpsyg.2026.1829093

**Published:** 2026-07-07

**Authors:** Chunjing Yang, Qijun Hu, Lin Fu, Yuanlong Jiang, Zhi Zhu, Han Ding, Wei Wang, Yun Tao, Xie Ma

**Affiliations:** 1 Faculty of Education, Yunnan Normal University, Kunming, China; 2 School of Psychology, Yunnan Normal University, Kunming, China

**Keywords:** musical training, music-based rhythmic movement training, reading ability, working memory, children from multilingual backgrounds

## Abstract

**Introduction:**

Reading ability plays an important role in children's cognitive and emotional development and school adjustment. Although musical training has been associated with children's reading-related abilities, the cognitive mechanisms underlying this relationship remain insufficiently understood. This study examined the relationship among musical training, working memory, and reading ability in children from multilingual backgrounds in frontier regions.

**Methods:**

A combined cross-sectional comparison and intervention study design was used. Study 1 compared children with musical training and children without musical training and examined the mediating role of working memory in the relationship between musical ability and reading-related outcomes. Study 2 implemented a 12-week music-based rhythmic movement training program consisting of 20 sessions among lower-grade children without prior musical training.

**Results:**

In Study 1, children with musical training performed better than children without musical training in musical ability, working memory, and some reading tasks. Working memory partially mediated the relationship between musical ability and antonym judgment and fully mediated the relationship between musical ability and sentence proposition judgment. In Study 2, children in the music intervention group showed improvements in musical ability, working memory accuracy, antonym judgment, and sentence proposition judgment.

**Discussion:**

These findings provide evidence from both an associational pathway and an intervention context, suggesting that working memory may represent a possible cognitive pathway linking musical training to the development of some reading-related abilities. Music-based rhythmic movement training may provide a feasible approach for supporting reading-related development among children from multilingual backgrounds in frontier regions.

## 1 Introduction

In modern society, reading is an important means of acquiring knowledge, and the development of reading ability is closely related to children's personal growth and mental health. Reading ability refers to the capacity to recognize words, access meaning, and construct text-level information through linguistic knowledge and prior experience (Perfetti, 2007). According to the stage model of reading development, elementary school children are in an important period of transition from “learning to read” to “reading to learn” (Chall, 1983; Fitzgerald and Shanahan, 2000). Reading ability is closely associated with children's cognitive and socio-emotional development (Sullivan and Brown, 2015; Yu et al., 2023). Children with relatively poor reading ability may experience greater academic frustration and difficulties in school adjustment (Morgan et al., 2008), and may also be at increased risk of mental health difficulties, including low self-esteem and academic anxiety (Fishstrom et al., 2024). For children in China's frontier regions, reading development is embedded in a more complex multilingual context. In this study, multilingual background refers to the linguistic ecology in which children are exposed to Mandarin Chinese, local Chinese dialects, and ethnic minority languages, such as Dai, Yi, and Hani. Accordingly, “children from multilingual backgrounds” refers to children who are exposed to or use two or more languages or dialects across family, community, and school settings. This definition follows the functional perspective in bilingual and multilingual research, which emphasizes that bilingual or multilingual individuals do not necessarily have balanced proficiency across languages, but may use two or more languages or dialects in everyday life (Grosjean, 2013). Such multilingual backgrounds may shape children's reading development through cross-linguistic transfer, differences in language input, and shifts across language-use contexts, resulting in reading-development characteristics that differ from those of monolingual children (Babayigit et al., 2022; Li et al., 2020; Sun et al., 2025). Therefore, multilingual background is considered a key contextual feature of the child population examined in this study. Focusing on children from multilingual backgrounds may deepen our understanding of individual differences in reading development and has practical significance for promoting academic adjustment, mental health, and educational equity in frontier regions.

As a vital medium for human emotional communication, music is deeply embedded in children's daily lives. Musical training, in particular, has been shown to support the development of children's language and reading abilities. Previous studies have reported that musical training of different durations may benefit several aspects of children's reading-related performance. For example, compared with conventional instruction, a six-week digital rhythm training program significantly improved children's reading fluency (Zanto et al., 2024). Children who participated in a 20-week musical training program showed significant gains in phonological awareness (Degé and Schwarzer, 2011), and 6 months of musical training significantly enhanced children's ability to read inconsistent words (Moreno et al., 2009). Beyond behavioral evidence, similar findings have also been reported at the neural level. Children who participated in a two-year community music program showed enhanced neurophysiological discrimination of similar speech syllables, suggesting improved neural encoding of speech sounds in the auditory brainstem pathway (Kraus et al., 2014). According to the OPERA (overlap, precision, emotion, repetition, attention) hypothesis, music and language share processing mechanisms for acoustic features, and the demands of musical activities on precise acoustic processing, emotional engagement, repetition, and attentional focus may promote neuroplasticity in speech-processing systems (Patel, 2011). Such music-induced plasticity may strengthen children's neural encoding of speech sounds and provide a possible foundation for language acquisition and reading development. Overall, existing studies suggest a close association between musical training and children's reading development across behavioral and neural levels.

Despite growing evidence for the potential benefits of musical training for children's reading development, several gaps remain in the existing literature. First, most previous studies have focused on children in ordinary school or community samples from Western alphabetic-language contexts (Eccles et al., 2021; Zanto et al., 2024), whereas evidence from Chinese-speaking children, particularly children from multilingual backgrounds in frontier regions, remains limited. Second, existing studies have mainly examined phonological awareness, phonological discrimination, early literacy skills, word-reading accuracy, or general reading fluency (Cancer and Antonietti, 2022; Eccles et al., 2021). However, reading ability also involves multiple levels of processing, including lexical-semantic representation, sentence-level meaning integration, and fluent sentence-level processing (Perfetti, 2007; Perfetti and Stafura, 2014; Fuchs et al., 2001). Third, phonological awareness has often been considered a key mechanism through which musical training may support reading development (Gordon et al., 2015), but this explanatory pathway remains debated (Sala and Gobet, 2020; Schellenberg and Lima, 2024). In a second-language reading context, music perception was associated with ESL silent reading comprehension, and this association was partly mediated by auditory working memory, suggesting that working memory may also be involved in the link between music perception and reading comprehension (Zhang, 2025). According to the experience-driven hypothesis, long-term musical training may not only affect music-related abilities, but may also transfer to non-musical domains, including cognitive abilities (Patel, 2014). Meta-analytic evidence has further suggested that musical training can improve higher-order cognitive functions in children, including working memory (Lu et al., 2025). In addition, long-term musical training may promote cognitive development by enhancing neural plasticity in relevant brain regions (Habibi et al., 2018). Taken together, these findings suggest that the mechanisms linking musical training to reading ability may not be limited to phonological awareness. The potential effects of musical training on cognitive abilities, particularly working memory, may represent another important pathway through which musical training supports children's reading development.

Accordingly, the present study aimed to examine the mediating role of working memory in the association between musical training and children's reading ability. Working memory (WM) refers to a limited-capacity system for temporarily storing and manipulating information during complex cognitive activities (Baddeley, 2003), and is considered an important foundation for higher-order cognition. Previous studies have shown that individuals with musical training generally perform better than those without musical training on short-term memory and working memory tasks (Talamini et al., 2017). Longitudinal and intervention studies with children have also reported associations between musical training and working memory development (Saarikivi et al., 2019; Nie et al., 2022). Reading development is supported by both domain-general cognitive processes and domain-specific reading-related skills (Cutting and Scarborough, 2006). Domain-general processes refer to cognitive abilities that are not specific to a single domain and operate across multiple task contexts, such as inhibitory control, cognitive shifting, and working memory. Among these processes, working memory has been identified as a robust predictor of reading across both alphabetic and logographic writing systems, suggesting cross-linguistic consistency in its role in reading development (Peng et al., 2018). Working memory is commonly conceptualized as comprising three components: the phonological loop, the visuospatial sketchpad, and the central executive system (Baddeley, 2000, 2003). Among these components, the central executive system has been suggested to play a particularly important role in reading ability (Swanson, 1999). The potential involvement of working memory in musical training may be related to the cognitive demands of musical activities. Musical training is not limited to auditory discrimination; rather, it requires children to maintain pitch, rhythm, and melodic sequences during continuous musical input, while also engaging in updating, comparison, prediction, motor coordination, and multisensory integration (Miendlarzewska and Trost, 2014; Slevc et al., 2016; Nie et al., 2022). Active musical activities, such as instrumental performance and rhythmic movement training, further require the coordination of auditory, visual, bodily, and motor processes (Miendlarzewska and Trost, 2014). These processes may therefore involve the central executive system. Regarding the assessment of working memory, n-back tasks have been widely used in working memory research. Among them, low-load one-back tasks are considered suitable for school-aged children because of their relatively low task difficulty (Pelegrina et al., 2015). Therefore, the present study selected working memory as a key domain-general cognitive factor and used performance on a one-back task as an indicator of children's working memory, with the aim of examining its mediating role in the association between musical training and children's reading ability.

The Simple View of Reading (SVR) proposes that reading ability is primarily composed of two components: word decoding and language comprehension. Word decoding emphasizes the accurate recognition of written symbols and their mapping onto phonological forms, whereas language comprehension involves the understanding and construction of word meanings, sentence structures, and text-level meaning (Gough and Tunmer, 1986; Hoover and Gough, 1990). Building on the SVR, Scarborough's Reading Rope model further decomposes reading ability into multiple strands within the language-comprehension and word-recognition systems, emphasizing that skilled reading emerges from the gradual integration of vocabulary knowledge, language structures, word recognition, and automatic reading processes (Scarborough, 2001). Based on these theoretical perspectives, the present study defined reading ability as children's integrated reading performance in lexical-semantic comprehension, sentence-level meaning integration, and automatic word recognition during written-language processing. Specifically, reading ability was operationalized through three interrelated components. The antonym judgment task assessed children's understanding of word meanings and lexical-semantic relations, reflecting the lexical-semantic comprehension component of reading ability. This task required children to retrieve word meanings and discriminate semantic relations in order to judge whether two words formed an antonymic relation (Frishkoff et al., 2008). The sentence proposition judgment task assessed children's ability to judge whether the content of a sentence was reasonable or true, reflecting the sentence-level meaning integration component of reading ability. This task required children to integrate syntactic structure and semantic information on the basis of word recognition and then determine whether the proposition expressed by the sentence was valid (Zhao et al., 2019). The sentence oral reading fluency task assessed children's ability to read continuous written materials aloud rapidly and accurately, reflecting automatic word recognition and reading-processing efficiency. In this task, children were required to read all materials as quickly and accurately as possible, and the number of correctly read Chinese characters per minute was used as the index (Yeung et al., 2016). Together, these three subtests operationalized children's reading ability at three levels: lexical-semantic comprehension, sentence-level meaning integration, and automatic word recognition. In line with this operational definition, the task materials were adapted from a study on the reading ability of children from multilingual backgrounds in China (Li, 2008), in order to improve the appropriateness of the test content and to better match the language experience and difficulty level of children from multilingual backgrounds.

In summary, the present research conducted two studies to systematically examine the relationships among musical training, working memory, and reading ability in children from multilingual backgrounds in frontier regions. Study 1 compared children with and without musical training on a one-back task and reading ability tasks, in order to examine the association between musical training and children's reading ability, as well as the potential mediating role of working memory. We hypothesized that children with musical training would perform better than children without musical training on working memory and reading ability tasks (H1a), and that working memory would mediate the relationship between musical ability and reading ability (H1b). Building on Study 1, Study 2 further implemented a longitudinal intervention with children without prior musical training in an educational practice setting, in order to examine whether musical training could improve children's working memory and reading ability. We hypothesized that children who received musical training would show improvements in working memory and reading ability after the intervention (H2). The findings were expected to clarify the cognitive mechanisms through which musical training is associated with reading ability in children from multilingual backgrounds in frontier regions, and to provide empirical evidence for educational practice.

## 2 Study 1: testing the pathway through which musical training influences children's reading ability via working memory

### 2.1 Methods

#### 2.1.1 Participants

This study adopted a grade-stratified recruitment strategy, with different primary school stages serving as sampling strata. Children were classified into a musically trained group and a non-musically trained group according to whether they had prior musical training experience. Children in the musically trained group were included if they had received more than 1 year of formal musical training, mainly including instrumental performance and vocal singing, and practiced regularly for at least 5 h per week. Children in the non-musically trained group had not participated in any extracurricular musical training other than regular school music classes. The required sample size was estimated using G^*^Power 3.1 (Faul et al., 2009). Because the main hypothesis of Study 1 focused on the main effect of musical training, the sample size calculation was based on a design with six groups formed by grade level and musical training experience, a numerator degree of freedom of df = 1, a significance level of α = 0.05, and a statistical power of 1 – β = 0.80. The estimated minimum total sample size was 128 participants. A total of 137 children were eventually recruited. The lower-grade group (Grades 1–2) included 41 children (age: 7.24 ± 0.49 years), with 21 children in the musically trained group (10 boys, 11 girls) and 20 children in the non-musically trained group (5 boys, 15 girls). The middle-grade group (Grades 3–4) included 48 children (age: 8.58 ± 0.50 years), with 28 children in the musically trained group (12 boys, 16 girls) and 20 children in the non-musically trained group (7 boys, 13 girls). The upper-grade group (Grades 5–6) included 48 children (age: 10.32 ± 0.47 years), with 25 children in the musically trained group (10 boys, 15 girls) and 23 children in the non-musically trained group (11 boys, 12 girls).

All participants had no history of diagnosed neurological or developmental disorders and had normal or corrected-to-normal vision and hearing. Before the experiment, the experimenter provided guardians with an informed consent form describing the purpose of the study, the testing and experimental procedures, and the anonymized processing of data. After the study had been explained and all questions had been answered, written informed consent was obtained from the guardians. The researchers also explained the study to the children in child-friendly language and confirmed their voluntary willingness to participate. Children were informed that they could withdraw from the study at any time without providing a reason. After completing the testing and experimental tasks, the children received a notebook as a token of appreciation. The study protocol was reviewed and approved by the Ethics Committee of the Faculty of Education, Yunnan Normal University.

#### 2.1.2 Measures

##### 2.1.2.1 Musical ability measure

To ensure the validity of the grouping of children with and without musical training, the abbreviated version of the Montreal Battery of Evaluation of Musical Abilities (MBEMA) was used to assess children's musical ability (Peretz et al., 2013). The materials are openly available at https://peretzlab.ca/. The abbreviated MBEMA is a standardized measure of musical ability for children. It covers core dimensions of musical ability, including melody, rhythm, and musical memory. This measure has been validated in samples from different cultural backgrounds, including Chinese children, and has shown cross-cultural applicability. Compared with the full version, the abbreviated version contains fewer items and requires less administration time. It can reduce fatigue and attentional load in children. Therefore, it is suitable for screening musical ability, verifying group classification, and assessing training effects among primary school children in school settings. The assessment consisted of three subtests: the melodic test, the rhythm test, and the memory test. Each subtest included two practice trials and 20 formal trials. Thus, the three subtests included 60 formal trials in total. In each trial, children judged whether the comparison stimulus was the same as the target stimulus. Each subtest included 10 same trials and 10 different trials. Correct responses were scored as 1, and incorrect responses were scored as 0. The scores of the three subtests were summed to obtain the total musical ability score. The total score ranged from 0 to 60. All participants completed the task using a specially designed experimental system. Auditory stimuli were presented through headphones connected to a tablet. Before the task began, instructions were displayed in black text on a white background. After a beep, two musical stimuli were presented with a brief interval. Children touched “√” or “ × ” on the screen to judge whether the two stimuli were the same. The tablet automatically recorded responses for each item. All formal trials were presented in a random order.

##### 2.1.2.2 Working memory task

**Figure 1:**
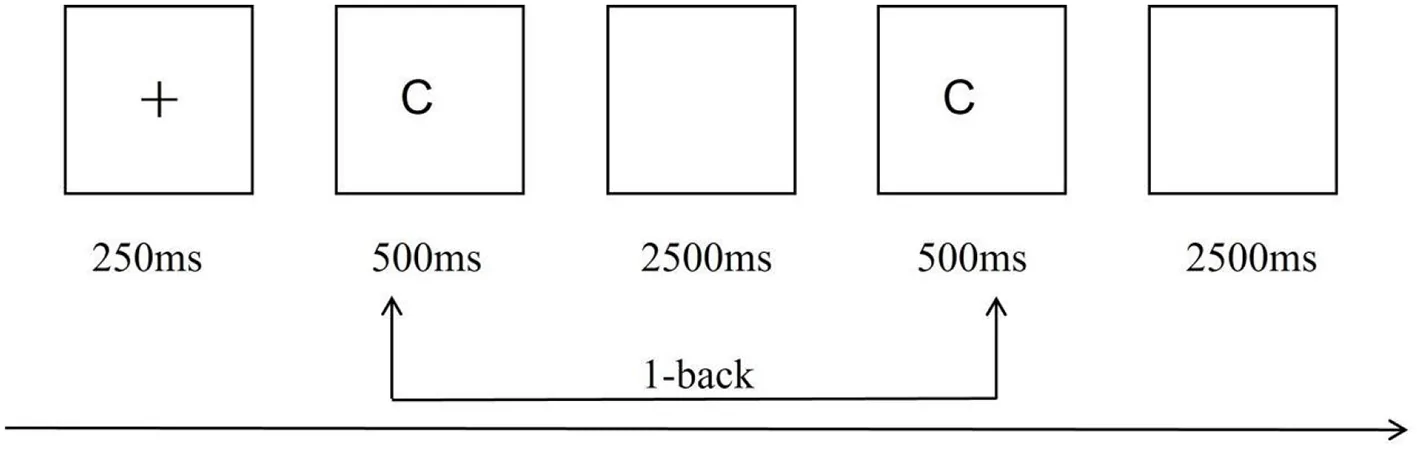
Experimental procedure of the one-back working memory task.

The working memory task used eight English letters as stimuli: C, D, G, K, P, Q, T, and V. This set of letters has been used as materials for N-back tasks in previous research on musical ability and executive function (Slevc et al., 2016). The same letter set has also been used to assess updating ability in a Chinese study on musical experience and executive function (Wang et al., 2019). Therefore, before the formal test, the experimenter confirmed that the children could recognize these letters. The letters were then used as stimuli in the working memory task. Compared with digits, words, or meaningful materials, English letters have relatively low semantic load. Their use may reduce the potential influence of word-meaning processing, numerical fluency, and mathematical experience on working memory performance. In addition, these eight letters are visually distinguishable. This feature may help reduce non-target errors caused by stimulus confusion. Therefore, this study used these letters as task materials to assess children's working memory updating ability more specifically. Children were asked to judge as quickly and accurately as possible whether the letter presented on the screen was the same as the immediately preceding stimulus. Each stimulus was presented in white at the center of the screen against a black background. Each trial began with a fixation point presented for 250 ms, followed by a letter stimulus presented for 500 ms. The response window was 2,500 ms. The task included both practice trials and formal trials. The formal task consisted of one block with 20 stimuli, including 6 target stimuli and 14 non-target stimuli. Before the formal task, children completed practice trials using the same rules as the formal task. During practice, feedback was provided after each trial. A “√” or “ × ” was displayed at the center of the screen to indicate whether the response was correct or incorrect. Children completed at least eight practice trials. They proceeded to the formal task only after reaching an accuracy rate of 80%. If this criterion was not met, they continued practicing until the criterion was reached. The experimental procedure is shown in Figure 1.

##### 2.1.2.3 Reading ability tasks

The reading ability test included three tasks developed by (Li 2008): antonym judgment, sentence proposition judgment, and sentence oral reading fluency. (Li 2008) reading ability test was developed in the context of assessing reading ability among children from multilingual backgrounds in China. It was designed for primary school children across different grade levels. The task content and language materials were used by (Li 2008) with a sample of 448 Chinese children from multidialectal backgrounds in Grades 2, 4, and 6, and showed good applicability. Therefore, the test was considered contextually appropriate for children from multilingual backgrounds in China's frontier regions. Based on this test, the present study adapted the materials to improve the match between the test content and children's language experience, learning level, and task difficulty. Cultural adaptations were also made to better fit the cognitive level, language habits, and cultural context of children from multilingual backgrounds. Details of the adaptations are provided in Appendix 1. In the antonym judgment task, children judged whether two single-character words presented as a pair formed an antonymic relation, such as “long–short.” They responded “yes” or “no.” The task included 50 items, with an equal number of affirmative and negative trials. In the sentence proposition judgment task, children read simple written sentences and judged whether the sentence meaning was correct or reasonable, such as “He speaks fast, so he runs fast.” Each sentence contained no more than 14 Chinese characters, with an average length of 12 characters. The task included 50 items, with an equal number of affirmative and negative trials. In the sentence oral reading fluency task, children read aloud the sentences presented on the screen as quickly as possible. Sentence oral reading fluency was indexed by the average number of correctly read Chinese characters per minute. This task included 30 sentences. The complete materials for the reading ability test are provided in Appendices 2–4.

#### 2.1.3 Research design

A 2 (Group: musically trained group, non-musically trained group) × 3 (Grade level: lower-grade group, middle-grade group, upper-grade group) between-subjects design was used. Both group and grade level were between-subjects variables. The dependent variables were musical ability score, working memory accuracy, working memory reaction time, antonym judgment score, sentence proposition judgment score, and sentence oral reading fluency.

#### 2.1.4 Procedure

The experiment was conducted by five experimenters who had received standardized training. Stimuli were presented on HUAWEI MatePad M3 tablets with an 8.4-inch IPS screen, a resolution of 2,560 × 1,600 pixels, and a refresh rate of 60 Hz. Audio stimuli were presented through SENDEM headphones. The experiment was conducted in a quiet, separate room. To reduce fatigue effects and order effects, participants completed the tasks in three separate sessions, with an interval of at least two class periods between consecutive sessions. The three types of tasks were reading ability tasks, musical ability tasks, and the working memory task. Their order was counterbalanced across participants. The three task types were coded as A, B, and C. Participants were randomly assigned to one of three order groups: ABC, BCA, or CAB. The number of participants in each order group was approximately balanced.

The reading ability test lasted approximately 35 min and included the antonym judgment task, the sentence proposition judgment task, and the sentence oral reading fluency task. The order of the three reading tasks was counterbalanced across participants to control for task-order effects. The tasks were administered using a self-designed experimental system, with visual stimuli presented in black text on a white background. In the antonym judgment and sentence proposition judgment tasks, participants touched the left or right side of the screen to provide affirmative or negative responses. The system automatically recorded response time and accuracy, and all task items were presented in a random order. In the sentence oral reading fluency task, participants tapped the screen to start recording and timing, read the sentence aloud, uploaded the recording after finishing, and then proceeded to the next sentence. The reading materials were presented in a random order. The musical ability test lasted approximately 25 min and included rhythm, melody, and memory subtests. Items in each subtest were presented in a random order. The working memory task lasted approximately 25 min. Stimuli were presented in a random order, and response time and accuracy were recorded automatically.

#### 2.1.5 Data analysis

All data were analyzed using IBM SPSS Statistics 25.0. Analysis of variance (ANOVA) was used, and Bonferroni correction was applied.

### 2.2 Results

#### 2.2.1 Musical ability

**Figure 2:**
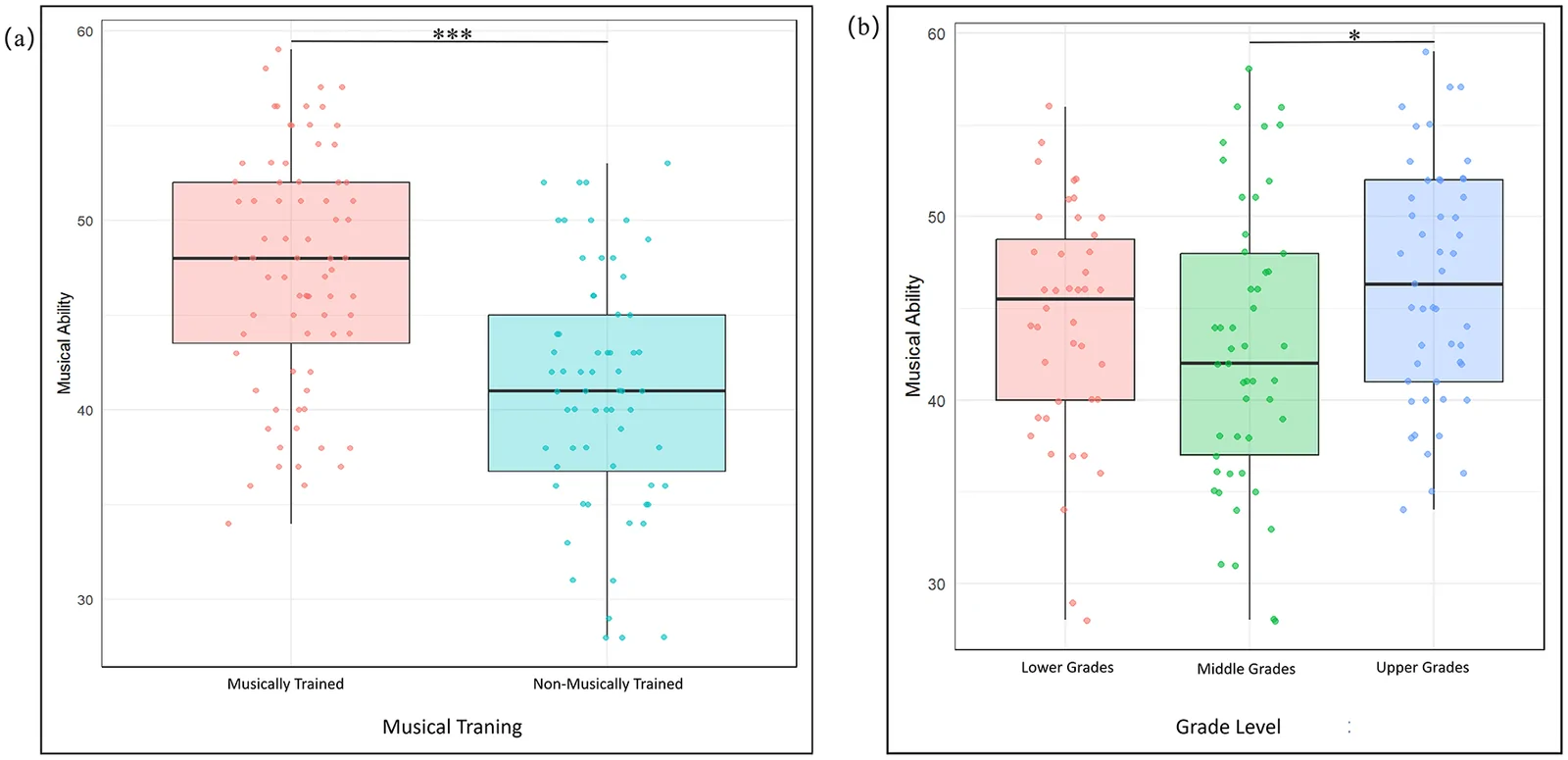
Musical ability scores by musical training experience and grade level. **(a)** Differences in musical ability scores between musically trained and non-musically trained children. **(b)** Differences in musical ability scores among lower-, middle-, and upper-grade children. *p < 0.05, **p < 0.01, ***p < 0.001.

A 2 (musical training: musically trained, non-musically trained) × 3 (grade level: lower grade, middle grade, upper grade) between-subjects analysis of variance (ANOVA) was conducted on the total musical ability score. The results showed a significant main effect of musical training, F _(1, 131)_ = 38.982, *p* < 0.001, $\eta_{p}^{2}$ = 0.229. Simple effects analysis showed that musically trained children (47.73 ± 0.72) had significantly higher musical ability scores than non-musically trained children (40.81 ± 0.77). The main effect of grade level was also significant, F _(2, 131)_ = 5.830, *p* = 0.004, $\eta_{p}^{2}$ = 0.082. Simple effects analysis showed that the middle-grade group (41.93 ± 0.89) had significantly lower musical ability scores than the upper-grade group (46.22 ± 0.88). The interaction between musical training and grade level was not significant, F _(2, 131)_ = 2.251, *p* = 0.109, $\eta_{p}^{2}$ = 0.033. See Figure 2 for details.

#### 2.2.2 Working memory

##### 2.2.2.1 Reaction time

**Figure 3:**
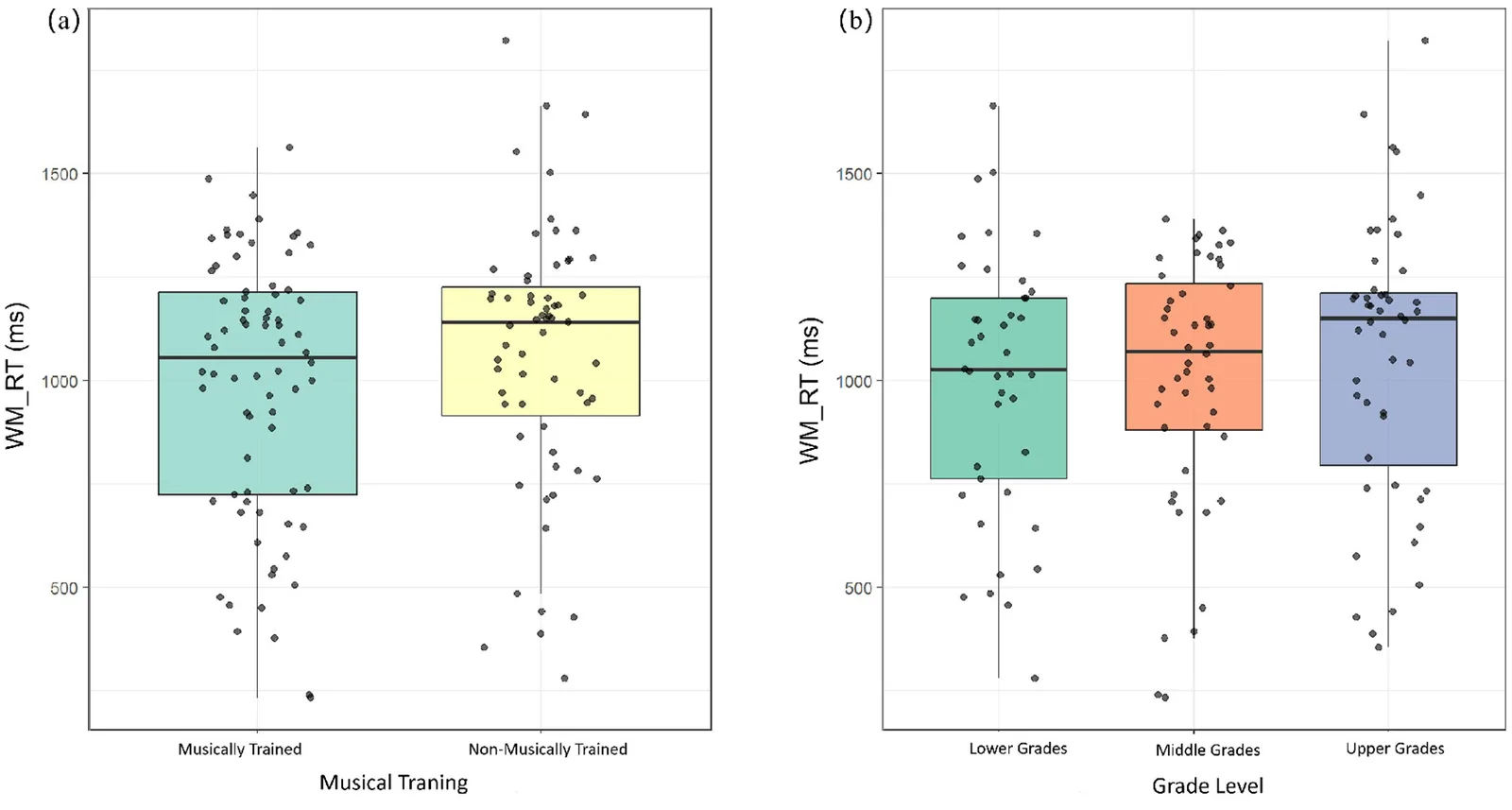
Working memory reaction time by musical training experience and grade level. **(a)** Differences in working memory reaction time between musically trained and non-musically trained children. **(b)** Differences in working memory reaction time among lower grades, middle grades, and upper grades. WM_RT = working memory reaction time.

A 2 (musical training: musically trained, non-musically trained) × 3 (grade level: lower grade, middle grade, upper grade) between-subjects ANOVA was conducted on reaction time in the working memory task. The results showed that the main effect of musical training was not significant, F _(1, 131)_ = 1.649, *p* = 0.201, $\eta_{p}^{2}$ = 0.012. The main effect of grade level was also not significant, F _(2, 131)_ = 0.710, p = 0.452, $\eta_{p}^{2}$ = 0.005. The interaction between musical training and grade level was not significant, F _(2, 131)_ = 0.865, *p* = 0.423, $\eta_{p}^{2}$ = 0.013. See Figure 3 for details.

##### 2.2.2.2 Accuracy

**Figure 4:**
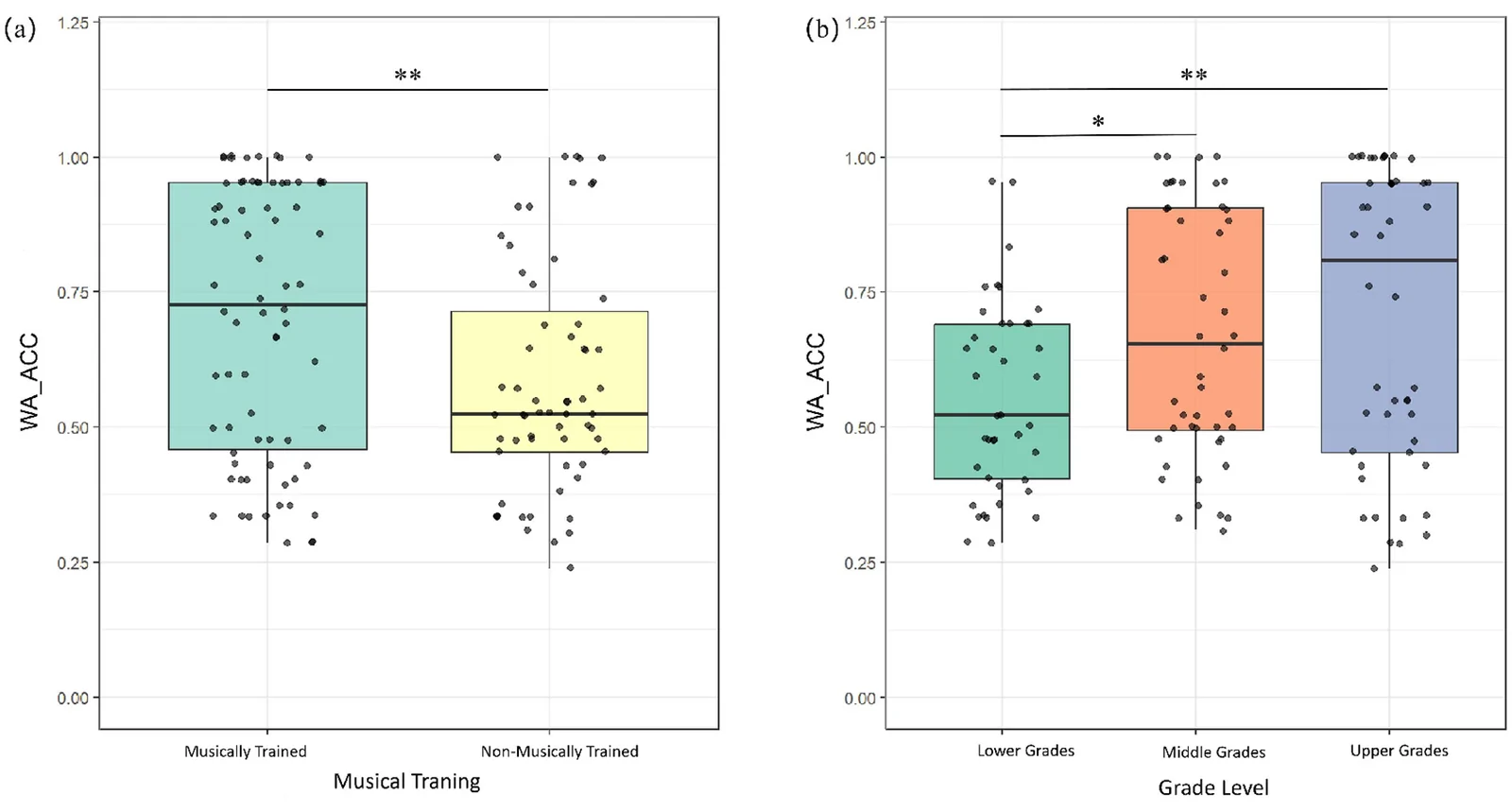
Working memory accuracy by musical training experience and grade level. **(a)** Differences in working memory accuracy between musically trained and non-musically trained children. **(b)** Differences in working memory accuracy among lower grades, middle grades, and upper grades. WM_ACC = working memory accuracy. *p < 0.05, **p < 0.01.

A 2 (musical training: musically trained, non-musically trained) × 3 (grade level: lower grades, middle grades, upper grades) between-subjects ANOVA was conducted on accuracy in the working memory task. The results showed a significant main effect of musical training, F _(1, 131)_ = 7.509, p = 0.007, $\eta_{p}^{2}$ = 0.054. Simple effects analysis showed that non-musically trained children had significantly lower accuracy (0.587 ± 0.029) than musically trained children (0.694 ± 0.026). The main effect of grade level was also significant, F _(2, 131)_ = 5.275, *p* = 0.005, $\eta_{p}^{2}$ = 0.075. Simple effects analysis showed that children in the lower grades had significantly lower accuracy than children in the middle grades (0.551 ± 0.035 vs. 0.669 ± 0.033, *p* = 0.017) and upper grades (0.551 ± 0.035 vs. 0.702 ± 0.033, p = 0.002). No other differences were significant (ps > 0.05). The interaction between musical training and grade level was not significant, F _(2, 131)_ = 1.337, *p* = 0.266, $\eta_{p}^{2}$ = 0.020. See Figure 4 for details.

#### 2.2.3 Reading ability

##### 2.2.3.1 Antonym judgment

**Figure 5:**
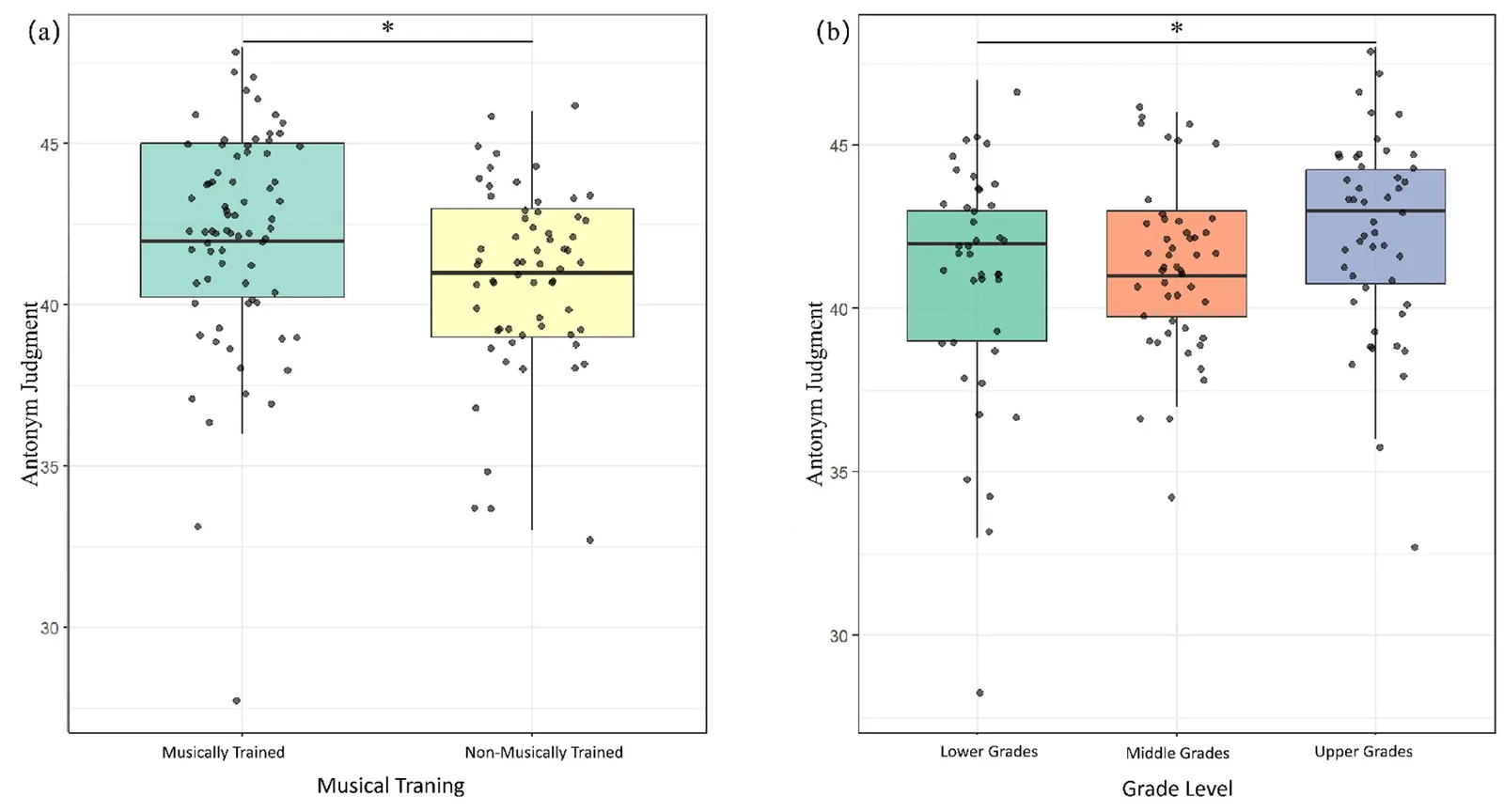
Antonym judgment scores by musical training experience and grade level. **(a)** Differences in antonym judgment scores between musically trained and non-musically trained children. **(b)** Differences in antonym judgment scores among lower grades, middle grades, and upper grades. *p < 0.05.

Correct responses in the antonym judgment task were scored as 1, and incorrect responses were scored as 0. A 2 (musical training: musically trained, non-musically trained) × 3 (grade level: lower grades, middle grades, upper grades) ANOVA was conducted on the total antonym judgment score. The results showed a significant main effect of musical training, F _(1, 131)_ = 5.506, *p* = 0.020, $\eta_{p}^{2}$ = 0.040. Simple effects analysis showed that musically trained children had significantly higher scores (42.093 ± 0.357) than non-musically trained children (40.859 ± 0.386). The main effect of grade level was marginally significant, F _(2, 131)_ = 2.683, *p* = 0.072, $\eta_{p}^{2}$ = 0.039. Although the overall grade-level effect did not reach conventional significance, pairwise comparison indicated that children in the upper grades had higher scores (42.319 ± 0.441) than children in the lower grades (40.945 ± 0.477, p = 0.036). The interaction between musical training and grade level was not significant, F _(2, 131)_ = 0.485, *p* = 0.617, $\eta_{p}^{2}$ = 0.007. See Figure 5 for details.

##### 2.2.3.2 Sentence proposition judgment

**Figure 6:**
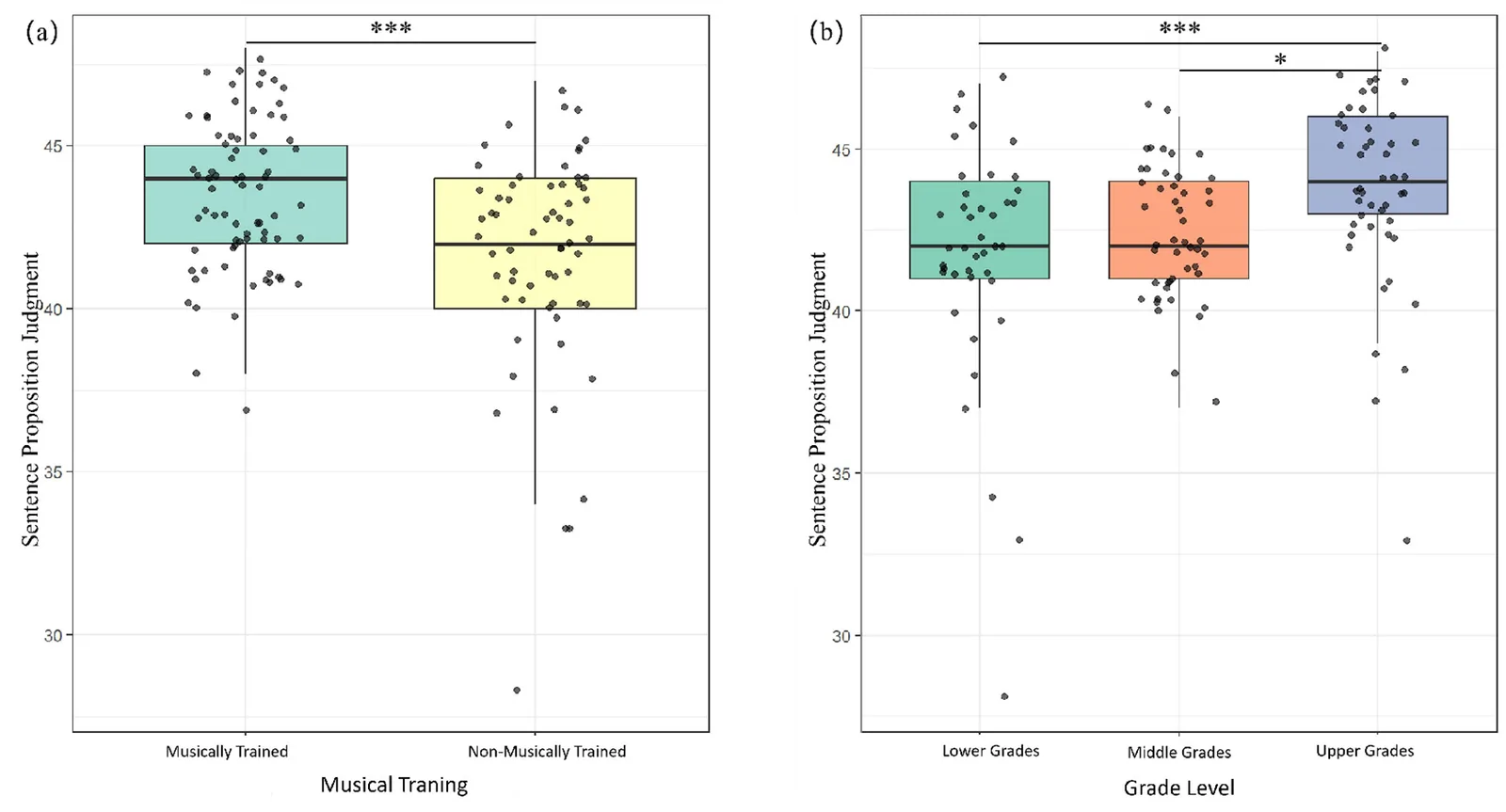
Sentence proposition judgment scores by musical training experience and grade level. **(a)** Differences in sentence proposition judgment scores between musically trained and non-musically trained children. **(b)** Differences in sentence proposition judgment scores among lower grades, middle grades, and upper grades. *p < 0.05, ***p < 0.001.

Correct responses in the sentence proposition judgment task were scored as 1, and incorrect responses were scored as 0. A 2 (musical training: musically trained, non-musically trained) × 3 (grade level: lower grades, middle grades, upper grades) ANOVA was conducted on the total sentence proposition judgment score. The results showed a significant main effect of musical training, F _(1, 131)_ = 16.578, *p* < 0.001, $\eta_{p}^{2}$ = 0.112. Simple effects analysis showed that musically trained children had significantly higher scores (43.494 ± 0.318) than non-musically trained children (41.588 ± 0.343). The main effect of grade level was also significant, F _(2, 131)_ = 6.867, *p* = 0.001, $\eta_{p}^{2}$ = 0.095. Simple effects analysis showed that children in the upper grades had significantly higher scores (43.703 ± 0.393) than children in the middle grades (42.296 ± 0.398, p = 0.013) and lower grades (41.625 ± 0.425, *p* < 0.001). The interaction between musical training and grade level was not significant, F _(2, 131)_ = 1.763, *p* = 0.176, $\eta_{p}^{2}$ = 0.026. See Figure 6 for details.

##### 2.2.3.3 Sentence oral reading fluency

**Figure 7:**
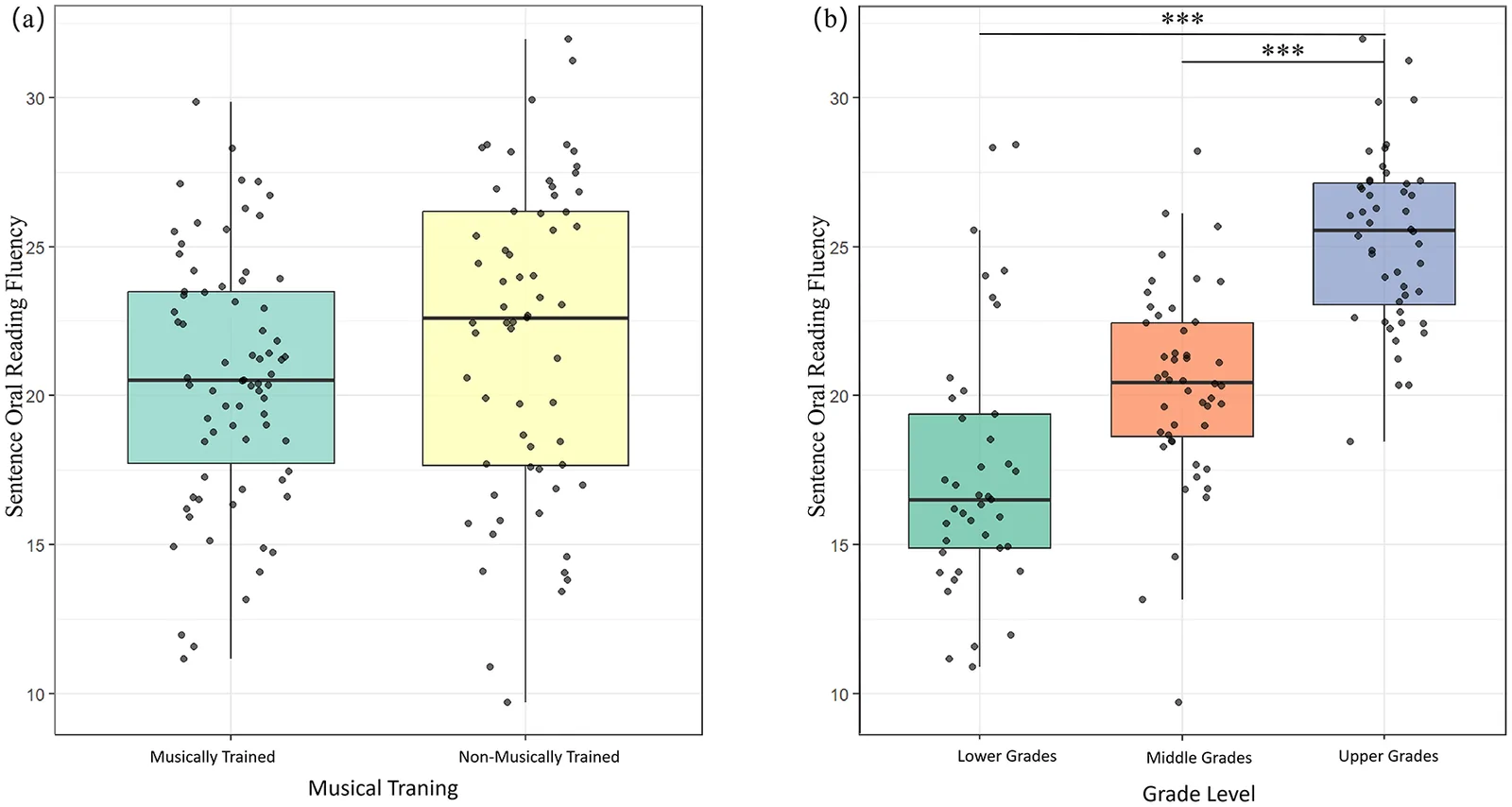
Sentence oral reading fluency by musical training experience and grade level**. (a)** Differences in sentence oral reading fluency between musically trained and non-musically trained children. **(b)** Differences in sentence oral reading fluency among lower grades, middle grades, and upper grades. ***p < 0.001.

A 2 (musical training: musically trained, non-musically trained) × 3 (grade level: lower grades, middle grades, upper grades) ANOVA was conducted on the average number of correctly read Chinese characters per minute. The results showed that the main effect of musical training was not significant, F _(1, 131)_ = 1.015, *p* = 0.315, $\eta_{p}^{2}$ = 0.008. The main effect of grade level was significant, F _(2, 131)_ = 56.725, *p* < 0.001, $\eta_{p}^{2}$ = 0.464. Simple effects analysis showed that children in the upper grades had significantly higher sentence oral reading fluency (25.297 ± 0.500) than children in the middle grades (20.379 ± 0.507, p < 0.001) and lower grades (17.498 ± 0.548, *p* < 0.001). Children in the middle grades also had significantly higher sentence oral reading fluency than children in the lower grades (*p* < 0.001). The interaction between musical training and grade level was not significant, F _(2, 131)_ = 0.613, *p* = 0.543, $\eta_{p}^{2}$ = 0.009. See Figure 7 for details.

#### 2.2.4 Path analysis

##### 2.2.4.1 Correlation analysis

**Figure 8:**
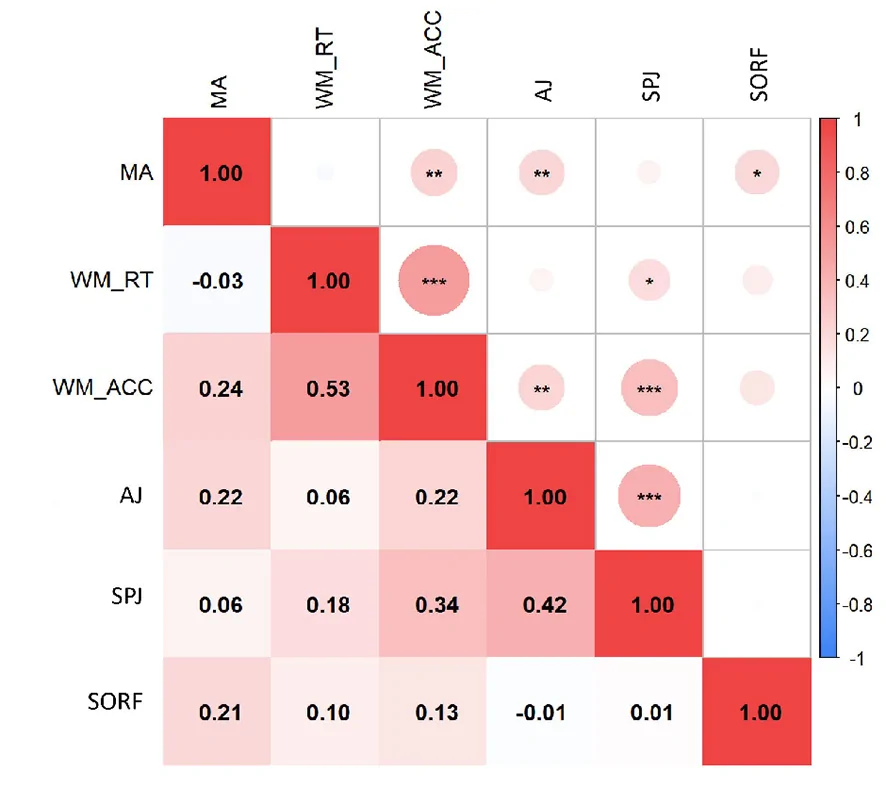
Correlation matrix among musical ability, working memory, and reading ability measures. MA = musical ability; WM_RT = working memory reaction time; WM_ACC = working memory accuracy; AJ = antonym judgment; SPJ = sentence proposition judgment; SORF = sentence oral reading fluency. *p < 0.05, **p < 0.01, ***p < 0.001.

Correlation analysis was conducted among musical ability, working memory reaction time, working memory accuracy, and the three dimensions of reading ability. The results showed that musical ability was positively correlated with working memory accuracy (r = 0.461, p < 0.001). Working memory accuracy was also positively correlated with sentence proposition judgment (*r* = 0.155, *p* = 0.036). The results are shown in Figure 8.

##### 2.2.4.2 Mediating role of working memory

**Figure 9:**
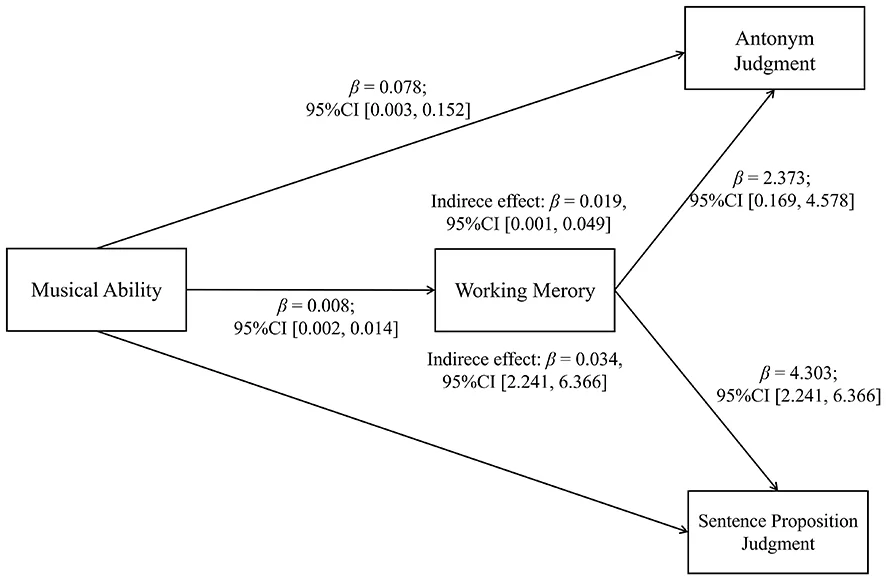
Mediation model of working memory in the relationship between musical ability and reading ability. MA = musical ability; WM = working memory; AJ = antonym judgment; SPJ = sentence proposition judgment. Path coefficients are shown in the figure.

To examine whether working memory mediated the relationship between musical ability and reading ability, mediation analysis was conducted using the PROCESS macro developed by Preacher and Hayes. Model 4 was used with the bootstrap method based on 5,000 resamples to test the mediating effect of working memory (Preacher and Hayes, 2008). The results showed that children's musical ability significantly and positively predicted working memory accuracy [β = 0.008, SE = 0.003, 95% CI (0.002, 0.014), excluding 0]. Working memory accuracy significantly and positively predicted children's antonym judgment [β = 2.373, SE = 1.115, 95% CI (0.169, 4.578), excluding 0] and sentence proposition judgment [β = 4.303, SE = 1.043, 95% CI (2.241, 6.366), excluding 0]. Musical ability also significantly and positively predicted children's antonym judgment [β = 0.078, SE = 0.038, 95% CI (0.003, 0.152), excluding 0], but did not significantly predict sentence proposition judgment [β = −0.009, SE = 0.035, 95% CI (−0.078, 0.061), including 0]. The mediating effect of working memory was significant [β = 0.047, SE = 0.022, 95% CI (0.004, 0.091), excluding 0], indicating that working memory accuracy partially mediated the relationship between musical ability and antonym judgment [β = 0.019, SE = 0.013, 95% CI (0.001, 0.050), excluding 0]. In addition, working memory accuracy fully mediated the relationship between musical ability and sentence proposition judgment [β = 0.034, SE = 0.016, 95% CI (0.009, 0.071), excluding 0], as shown in Figure 9.

### 2.3 Discussion

Study 1 mainly examined the relationships among musical training, working memory, and children's reading ability. The results showed that musically trained children had significantly higher musical ability scores than non-musically trained children, and that children in the upper grades had significantly higher musical ability scores than those in the middle grades. In terms of reading ability, musically trained children performed significantly better than non-musically trained children on antonym judgment and sentence proposition judgment. Sentence oral reading fluency did not show a significant difference between the two musical training groups, but showed significant differences across grade levels. To further clarify the cognitive pathways among musical training, working memory, and reading ability, mediation analysis was conducted using musical ability score, working memory accuracy, and scores on antonym judgment and sentence proposition judgment. The results showed that working memory partially mediated the relationship between musical ability and antonym judgment, and fully mediated the relationship between musical ability and sentence proposition judgment. Based on the group differences and mediating effects observed in Study 1, Study 2 further selected children without prior musical training to receive a musical training intervention, in order to examine changes in their musical ability, working memory, and reading ability before and after training.

## 3 Study 2: effects of music-based rhythmic movement training on children's working memory and reading ability

### 3.1 Methods

Music-based rhythmic movement training is a multisensory form of musical training (Juntunen and Sutela, 2023). The present study drew on the three core branches of Dalcroze Eurhythmics, namely eurhythmics, solfège, and improvisation (Johnson, 1993). The training also incorporated nursery rhymes, musical instruments, rhythmic movement activities, folk festival practices, and other culturally familiar materials from the everyday experiences of children from multilingual backgrounds. The training activities were designed to target several components of musical and cognitive processing. Body-based perception activities mainly involved rhythm processing and the updating of rhythm–movement sequences. Folk-song listening and singing activities involved melodic perception, pitch retention, and melodic sequence processing. Improvisational creation and performance required the temporary maintenance, online updating, and reproduction of musical materials, as well as prediction, inhibition, and coordination under rule-based constraints. Therefore, the melody, rhythm, and memory subtests of the short version of the MBEMA were considered appropriate for assessing the core musical processing abilities involved in the training. These subtests were also aligned with the demands of information maintenance, online updating, and central executive control embedded in the intervention. Building on Study 1, Study 2 implemented a 12 week music-based rhythmic movement training program consisting of 20 sessions to further examine whether musical training could support children's musical ability, working memory, and reading ability in an educational practice context.

#### 3.1.1 Participants

To control for the potential effects of age-related developmental differences and prior training experience on the intervention outcomes, participants were limited to children in the lower grades of primary school. Children were also required to have no prior systematic specialized training in either music or drawing before the intervention. An a priori sample size estimation was conducted for a 2 × 2 mixed-design ANOVA. With a medium effect size of 0.25, an alpha level of 0.05, and statistical power of 0.80, the required total sample size was estimated to be 34 participants. For Study 2, 60 children were newly recruited for the intervention study, with no overlap with the sample in Study 1. The music intervention group included 30 children (13 boys, 17 girls; age = 7.44 ± 0.50 years), and the drawing intervention group included 30 children (14 boys, 16 girls; age = 7.46 ± 0.51 years). Due to constraints in the real school setting, the present study used natural classroom-based cluster assignment within the school and allocated the intervention groups according to implementation feasibility. Similar cluster-based or real-classroom designs have been used in previous child music training intervention studies (Campbell et al., 2012; Jaschke et al., 2018). Baseline measurements showed no significant differences between the two groups in pretest performance, supporting the baseline comparability of the groups.

The experimenter provided guardians with an informed consent form that described the purpose of the study, the content of the musical training, and the procedures for anonymizing the data. After the study had been explained and questions had been answered, written informed consent was obtained from the guardians. The researchers explained the study to the children in age-appropriate, game-based language and confirmed their willingness to participate. During the training period, children's ongoing assent was verbally confirmed before each session, and they were allowed to withdraw from the study at any time without giving a reason. Children who completed the intervention and the pretest and posttest assessments received a certificate of participation and a pen as a token of appreciation. This study was approved by the Ethics Committee of the Faculty of Education, Yunnan Normal University.

#### 3.1.2 Study design

This study adopted a 2 (assessment time: pretest, posttest) × 2 (intervention group: Musical Training Group, Drawing Training Group) mixed design. Assessment time was the within-subject factor, and intervention group was the between-subject factor. The dependent variables were musical ability score, working memory accuracy, working memory reaction time, antonym judgment, sentence proposition judgment, and sentence oral reading fluency.

#### 3.1.3 Measures

The measures used in Study 2 were the same as those used in Study 1. These measures included the musical ability test, the working memory task, and the reading ability tasks.

#### 3.1.4 Procedure

Children completed the pretest before the intervention, using the same measures as those in Study 1. One week after the pretest, the children began the intervention program. The training was conducted outside regular school classes over a 12-week period. Each group was originally scheduled to receive 24 sessions. Due to school activities and holiday arrangements, 20 sessions were completed, with a completion rate of 83.3%. Each session lasted 55 min. The music intervention was jointly implemented by teachers with a professional background in music, inheritors of intangible cultural heritage, and parents with a professional background in music. Instructor competence was mainly confirmed through teaching certificates, music-related educational background, teaching experience, or certificates as inheritors of intangible cultural heritage. Before the intervention, centralized training was provided for instructors to clarify the intervention goals, course procedures, activity steps, and time allocation. During the intervention, basic supervision was conducted through pre-session preparation, post-session records, and occasional classroom observations to support consistent implementation across intervention conditions. All three core modules of the music intervention were completed according to the curriculum plan: eight sessions of body-based perception training, six sessions of folk-song listening and singing training, and six sessions of improvisational creation and performance. All planned module-specific sessions were completed. The posttest was administered within 1 week after the intervention.

An overview of intervention implementation and consistency across the two intervention conditions is presented in Table 1. The curriculum plan for music-based rhythmic movement training is shown in Table 2. Drawing training was used as an active control condition and was matched with the music intervention in training period, session duration, organizational format, and adult guidance. The detailed curriculum arrangement for drawing training is shown in Table 3.

**Table 1:** Overview of intervention implementation and consistency.

Intervention condition	Instructors	Frequency and duration	Planned sessions	Completed sessions	Completion rate	Completion of core modules	Implementation supervision
Music intervention	Music teachers, inheritors of intangible cultural heritage, and parents with professional music backgrounds	Twice per week, 55 min per session	24	20	83.3%	Body-based perception training: 8 sessions; folk-song listening and singing: 6 sessions; improvisational creation and performance: 6 sessions. All modules were completed.	Centralized training, pre-session preparation, post-session records, and occasional classroom observations
Drawing intervention	Art teachers, inheritors of intangible cultural heritage, and parents with professional art backgrounds	Twice per week, 55 min per session	24	20	83.3%	Intangible-cultural-heritage pattern drawing: 8 sessions; folk craft experience: 6 sessions; scene-based creative drawing: 6 sessions. All modules were completed.	Centralized training, pre-session preparation, post-session records, and occasional classroom observations

**Table 2:** Curriculum plan for music-based rhythmic movement training.

Training module	Instructors and sessions	Training content
Body Awareness Training (Eurhythmics)	Intangible Cultural Heritage Inheritors and Music Teachers; 8 Sessions	First Experience of Stamping Rhythm Movement — The Body as a Great Musical Instrument
		Stamping Rhythm Expert — Six Feet Dancing, Competing for the Hero
		Stamping Rhythm Dance Party — Folk Songs of Unity in the Circle
		Exploring the Rhythm of Hulusi (Gourd Flute)
		Hulusi Little Elf
		Tiger Hulusi's Great Adventure — The Mysterious Rhythm in the Forest
		Little Leopard Hulusi's Joyful Dance
		Joyful Torch Festival
Folk Music Listening and Singing Training (Sight-Singing and Ear Training)	Professional Parents and Music Teachers; 6 Sessions	Cup Rhythm Movement
		Balloon Clap Fun Park
		The Rhythm and Melody Around Me
		Melody Imitation Rhythm Challenge
		Guess the Instrument Timbre Rhythm Game
		Joyful Yi New Year
Improvisational Expression and Creation (Improvisational Composition)	Intangible Cultural Heritage Inheritors, Professional Parents, and Music Teachers	Big Gong Hulusi Rhythm Mobilization
		Big Gong Hulusi Rhythm Showdown
		My Intangible Cultural Heritage Rhythm Storytelling Session; 6 Sessions
		Melody and Rhythm Movement Magician
		Instrumental Rhythm Concerto
		Grand Celebration of Ethnic Unity

**Table 3:** Curriculum plan for drawing training.

Training module	Instructors and sessions	Training content
Intangible-cultural-heritage color patterns	Inheritors of intangible cultural heritage and art teachers; 8 sessions	The magical robe of the tie-dye elves: an initial experience of crayon-resist dyeing
		The magical robe of the tie-dye elves: the hexagonal flower-pattern challenge
		Dongba script treasure map: mysterious patterns in the mountain forest
		Dongba script treasure map: ethnic patterns in concentric circles
		Princess Kongque's colorful robe: exploring mysterious feather patterns
		Princess Kongque's colorful robe: exploring mysterious feather patterns
		Xiuxiu the Little Leopard's colorful robe: an initial experience of geometric patterns
		Xiuxiu the Little Leopard's colorful robe: a symmetrical-pattern collage challenge
Ethnic craft experience	Parents with professional art backgrounds and art teachers; 6 sessions	The secret layers of ink-wash mountains and forests: dry and wet painting techniques
		The secret of Jia Ma woodblock prints: magical mirror images in positive and negative forms
		Paper-cutting in the stone forest: ethnic patterns with near and far layers; The peony elf's sketching garden
		The peony elf's sketching garden
		The color magician's palette: a challenge with the three primary colors
		The pattern magician's transformation: redesigning patterns with symbolic elements
Ethnic craft experience	Parents with professional art backgrounds and art teachers; 6 sessions	Paper-cutting and folding of window flowers
		The guardian of blue-and-white porcelain: drafting curled grass patterns
		The guardian of blue-and-white porcelain: coloring light and dark layers
		The annual art exhibition: ethnic patterns made with color-block collage
		Yunnan memory: a poster competition
		My ethnic unity storytelling stage

#### 3.1.5 Data analysis

All data were analyzed using IBM SPSS Statistics 25.0. Repeated-measures ANOVA was used to compare changes in children's musical ability, working memory, and reading ability from pretest to posttest across the different intervention groups. Bonferroni correction was applied for multiple comparisons.

### 3.2 Results

#### 3.2.1 Musical ability

**Figure 10:**
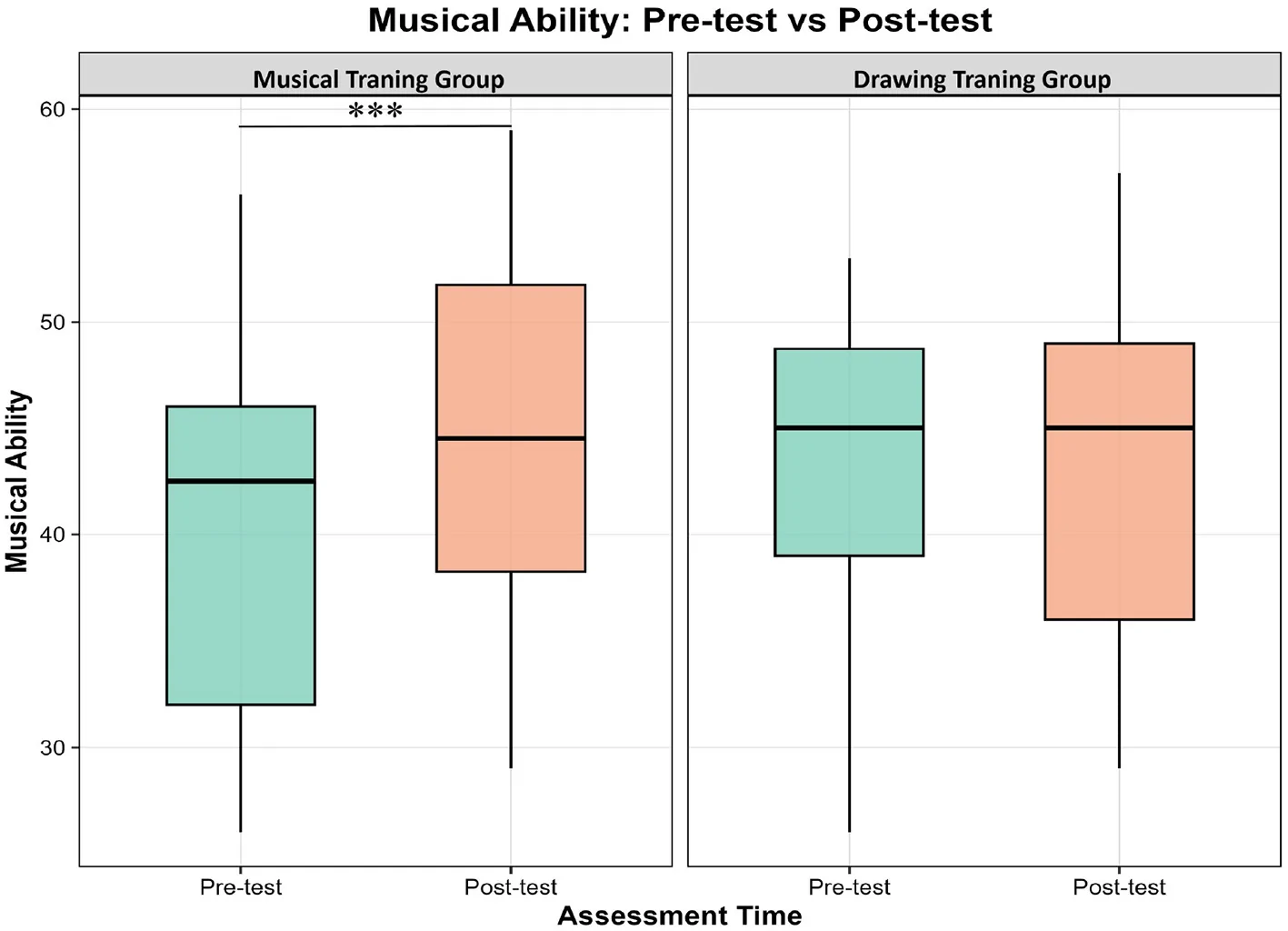
Musical ability scores from pretest to posttest by intervention group. The labels “Musical Training Group” and “Drawing Training Group” refer to the music intervention group and drawing intervention group, respectively. ***p < 0.001.

A 2 (assessment time: pretest, posttest) × 2 (intervention group: Musical Training Group, Drawing Training Group) repeated-measures ANOVA was conducted on the total musical ability score. The results showed a significant main effect of assessment time, F _(1, 58)_ = 9.832, *p* = 0.003, $\eta_{p}^{2}$ = 0.145. Simple effects analysis showed that musical ability scores were significantly higher at posttest (43.458 ± 1.082) than at pretest (41.500 ± 1.051). The main effect of intervention group was not significant, F _(1, 58)_ = 0.137, *p* = 0.713, $\eta_{p}^{2}$ = 0.002. The interaction between assessment time and intervention group was significant, F _(1, 58)_ = 5.679, *p* = 0.020, $\eta_{p}^{2}$ = 0.089. Simple effects analysis showed no significant difference in pretest scores between the Musical Training Group (40.333 ± 1.487) and the Drawing Training Group (44.000 ± 1.531), p = 0.272. In the Musical Training Group, the pretest score (40.333 ± 1.487) was significantly lower than the posttest score (44.000 ± 1.573), *p* < 0.001. In contrast, no significant pretest-to-posttest difference was observed in the Drawing Training Group, p = 0.597. See Figure 10 for details.

#### 3.2.2 Working memory

##### 3.2.2.1 Reaction time

**Figure 11:**
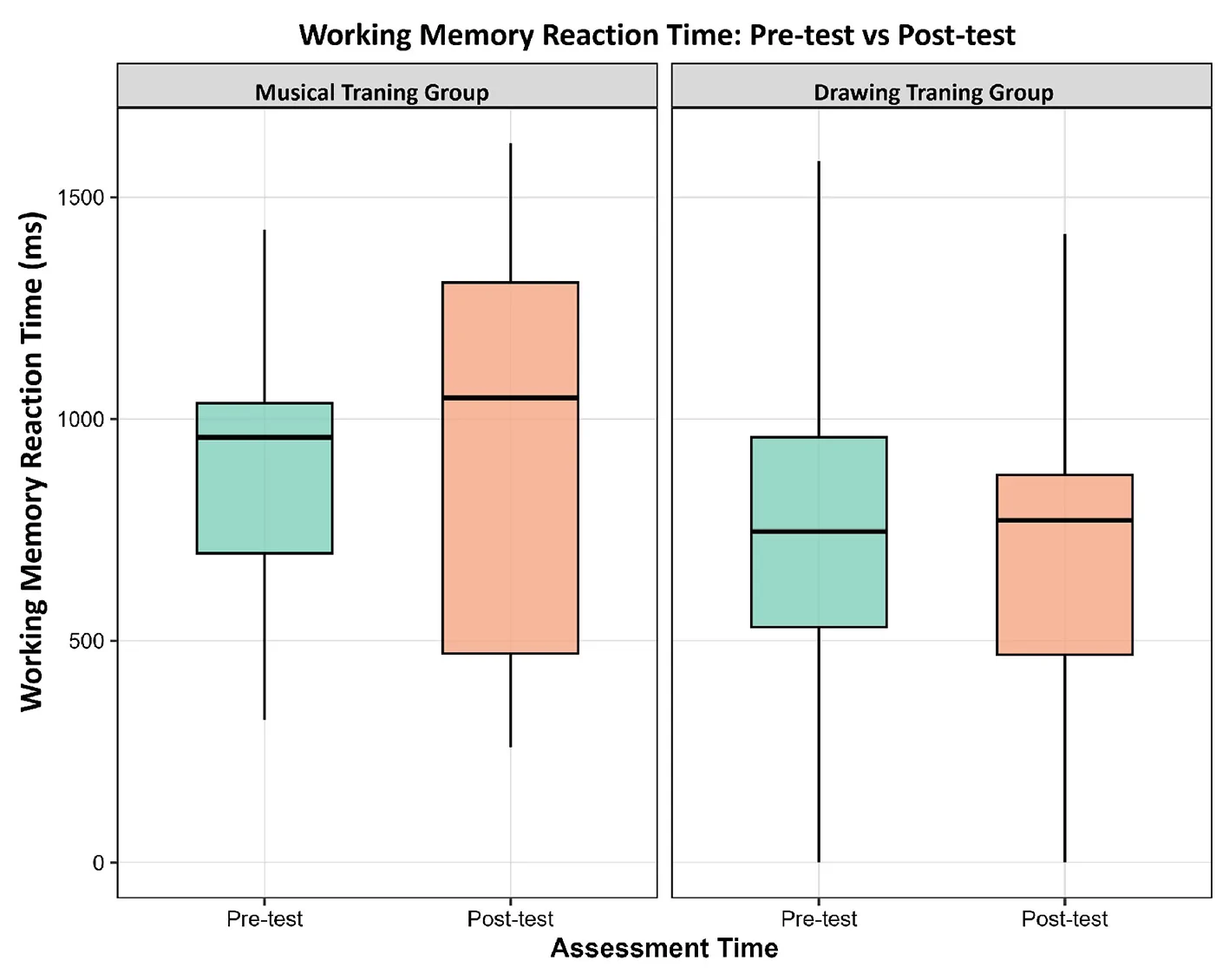
Working memory reaction time by assessment time and intervention group. Reaction time is presented in milliseconds. The labels “Musical Training Group” and “Drawing Training Group” refer to the music intervention group and drawing intervention group, respectively.

A 2 (assessment time: pretest, posttest) × 2 (intervention group: Musical Training Group, Drawing Training Group) repeated-measures ANOVA was conducted on working memory reaction time. The results showed that the main effect of assessment time was not significant, F _(1, 58)_ = 0.044, *p* = 0.834, $\eta_{p}^{2}$ = 0.001. The main effect of intervention group was significant, F _(1, 58)_ = 6.784, *p* = 0.012, $\eta_{p}^{2}$ = 0.105. Simple effects analysis showed that the Drawing Training Group had shorter reaction times (753.886 ± 383.241) than the Musical Training Group (928.921 ± 385.558). The interaction between assessment time and intervention group was not significant, F _(1, 58)_ = 1.126, *p* = 0.293, $\eta_{p}^{2}$ = 0.019. See Figure 11 for details.

##### 3.2.2.2 Accuracy

**Figure 12:**
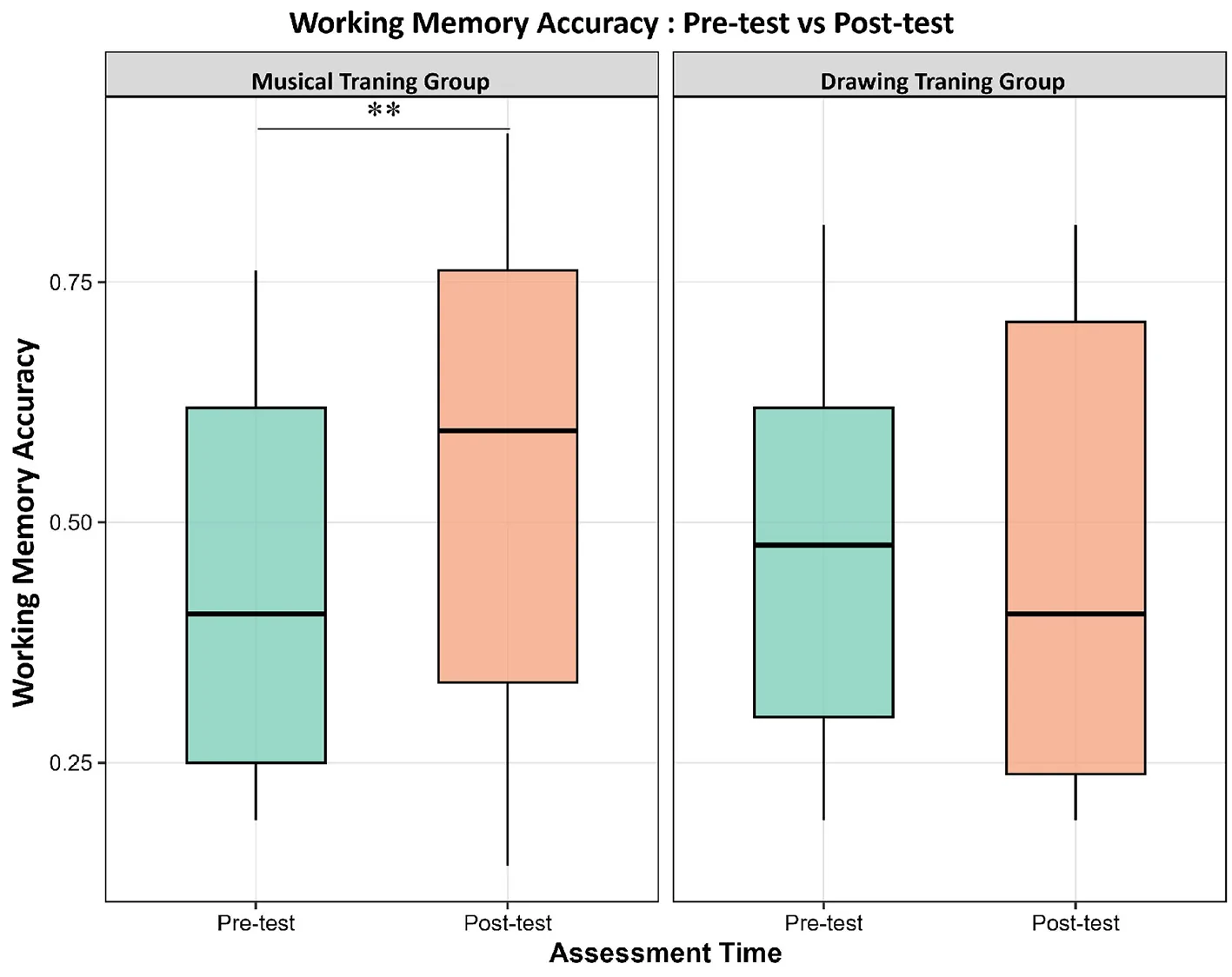
Working memory accuracy from pretest to posttest by intervention group. The labels “Musical Training Group” and “Drawing Training Group” refer to the music intervention group and drawing intervention group, respectively. **p < 0.01.

A 2 (assessment time: pretest, posttest) × 2 (intervention group: Musical Training Group, Drawing Training Group) repeated-measures ANOVA was conducted on working memory accuracy. The results showed a significant main effect of assessment time, F _(1, 58)_ = 4.367, *p* = 0.041, $\eta_{p}^{2}$ = 0.070. Simple effects analysis showed that working memory accuracy was significantly higher at posttest (0.515 ± 0.030) than at pretest (0.455 ± 0.024). The main effect of intervention group was not significant, F _(1, 58)_ = 0.164, *p* = 0.704, $\eta_{p}^{2}$ = 0.003. The interaction between assessment time and intervention group was significant, F _(1, 58)_ = 4.484, *p* = 0.039, $\eta_{p}^{2}$ = 0.072. Simple effects analysis showed that, in the Musical Training Group, working memory accuracy was significantly higher at posttest (0.554 ± 0.043) than at pretest (0.433 ± 0.034), *p* = 0.004. In contrast, no significant pretest-to-posttest difference was observed in the Drawing Training Group, *p* = 0.984. See Figure 12 for details.

#### 3.2.3 Reading ability

##### 3.2.3.1 Antonym judgment

**Figure 13:**
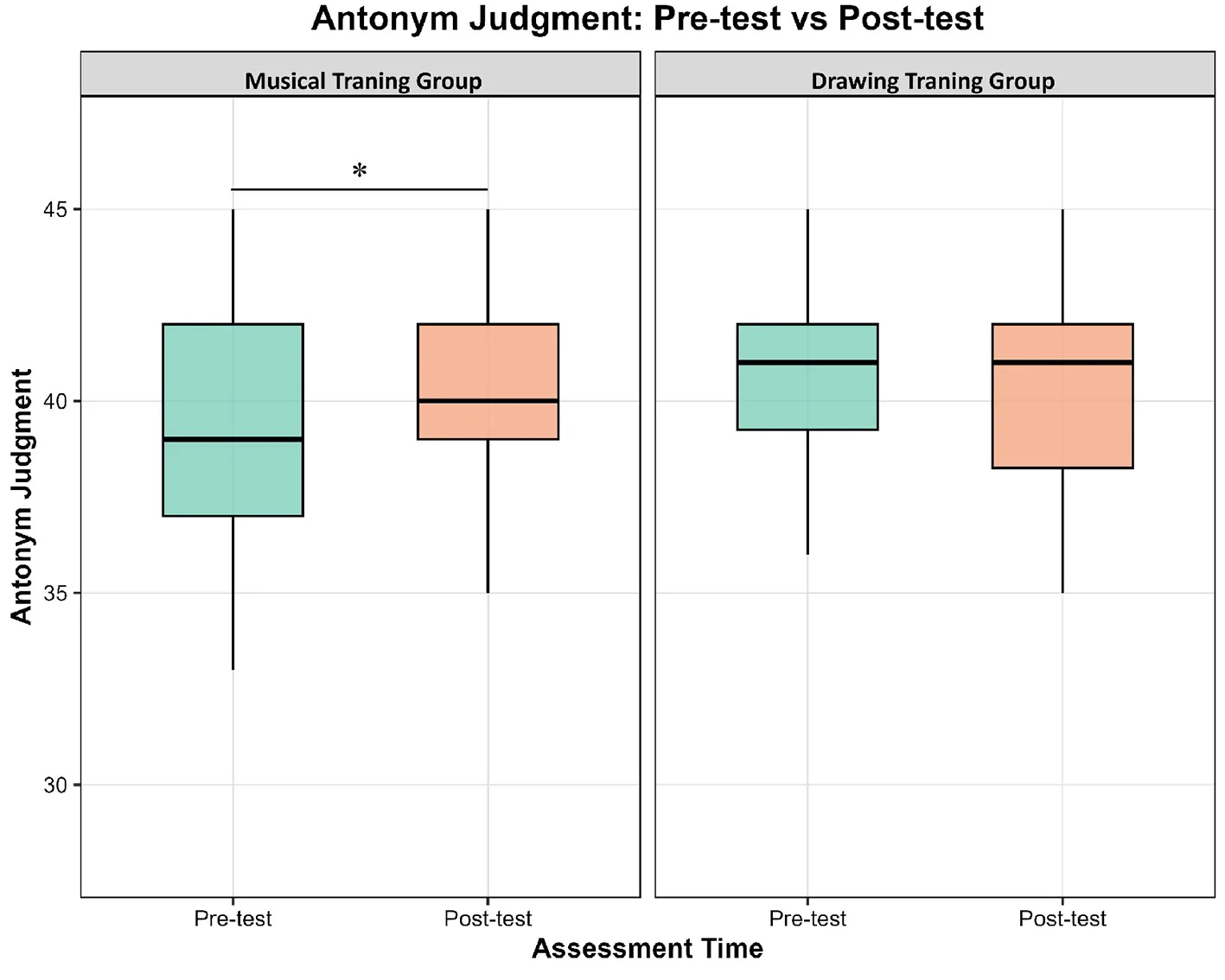
Antonym judgment scores from pretest to posttest by intervention group. The labels “Musical Training Group” and “Drawing Training Group” refer to the music intervention group and drawing intervention group, respectively. *p < 0.05.

For the antonym judgment task, correct responses were scored as 1, and incorrect responses were scored as 0. A 2 (assessment time: pretest, posttest) × 2 (intervention group: Musical Training Group, Drawing Training Group) repeated-measures ANOVA was conducted on the total antonym judgment score. The results showed that the main effect of assessment time was not significant, F _(1, 58)_ = 0.911, *p* = 0.344, $\eta_{p}^{2}$ = 0.015. The main effect of intervention group was not significant, F _(1, 58_) = 3.018, *p* = 0.088, $\eta_{p}^{2}$ = 0.049. The interaction between assessment time and intervention group was significant, F _(1, 58)_ = 6.228, *p* = 0.015, $\eta_{p}^{2}$ = 0.097. Simple effects analysis showed that, in the Musical Training Group, antonym judgment scores were significantly higher at posttest (40.200 ± 0.493) than at pretest (38.633 ± 0.623), p = 0.018. In contrast, no significant pretest-to-posttest difference was observed in the Drawing Training Group, *p* = 0.280. See Figure 13 for details.

##### 3.2.3.2 Sentence proposition judgment

**Figure 14:**
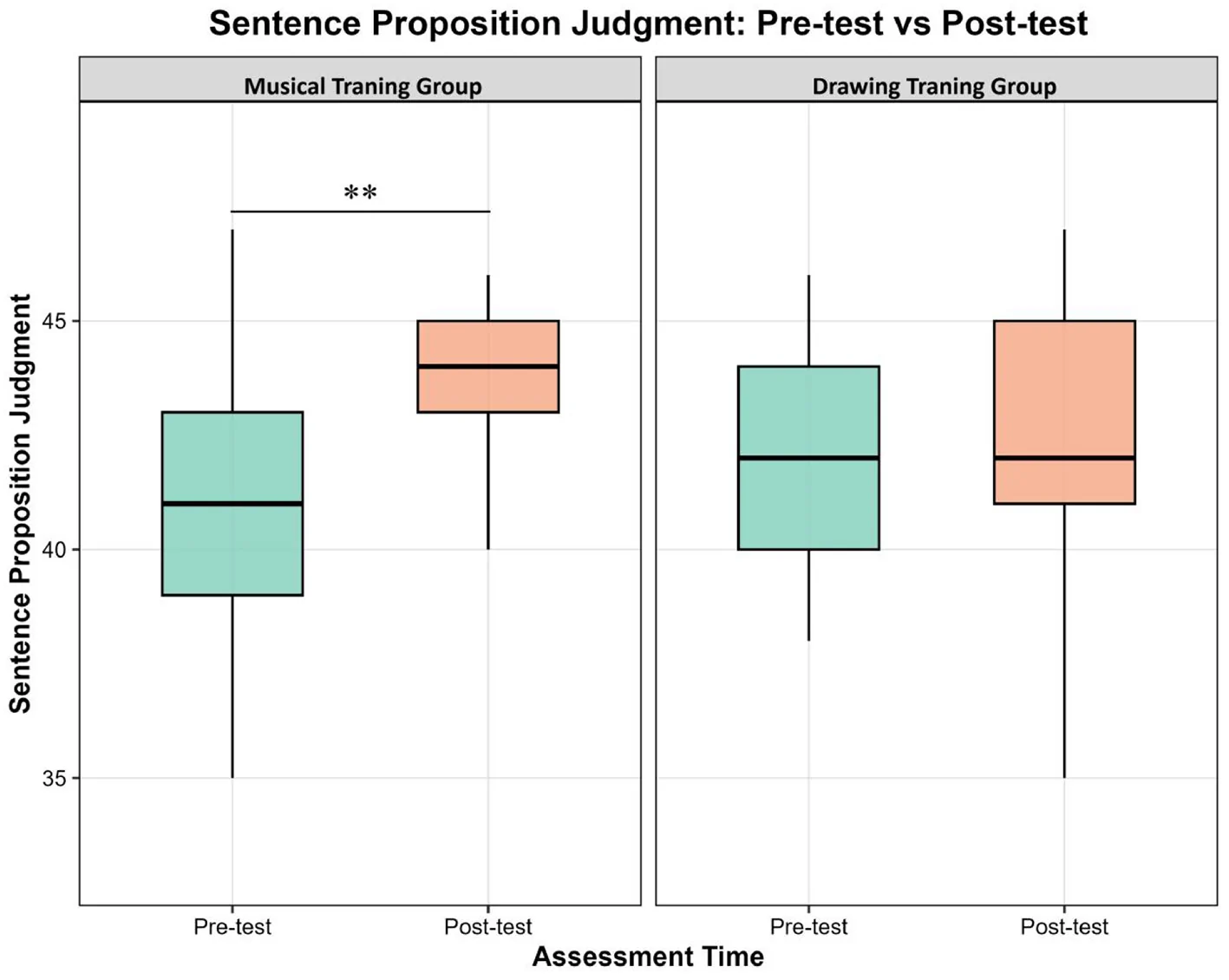
Sentence proposition judgment scores from pretest to posttest by intervention group. The labels “Musical Training Group” and “Drawing Training Group” refer to the music intervention group and drawing intervention group, respectively. **p < 0.01.

For the sentence proposition judgment task, correct responses were scored as 1, and incorrect responses were scored as 0. A 2 (assessment time: pretest, posttest) × 2 (intervention group: Musical Training Group, Drawing Training Group) repeated-measures ANOVA was conducted on the total sentence proposition judgment score. The results showed a significant main effect of assessment time, F _(1, 58)_ = 9.814, *p* = 0.003, $\eta_{p}^{2}$ = 0.145. The main effect of intervention group was not significant, F _(1, 58)_ = 0.243, *p* = 0.624, $\eta_{p}^{2}$ = 0.004. The interaction between assessment time and intervention group was significant, F _(1, 58)_ = 8.140, *p* = 0.006, $\eta_{p}^{2}$ = 0.123. Further simple effects analysis showed that, in the Musical Training Group, sentence proposition judgment scores were significantly higher at posttest (43.533 ± 0.606) than at pretest (39.967 ± 0.804), *p* < 0.001. In contrast, no significant pretest-to-posttest difference was observed in the Drawing Training Group, *p* = 0.844. See Figure 14 for details.

##### 3.2.3.3 Sentence oral reading fluency

**Figure 15:**
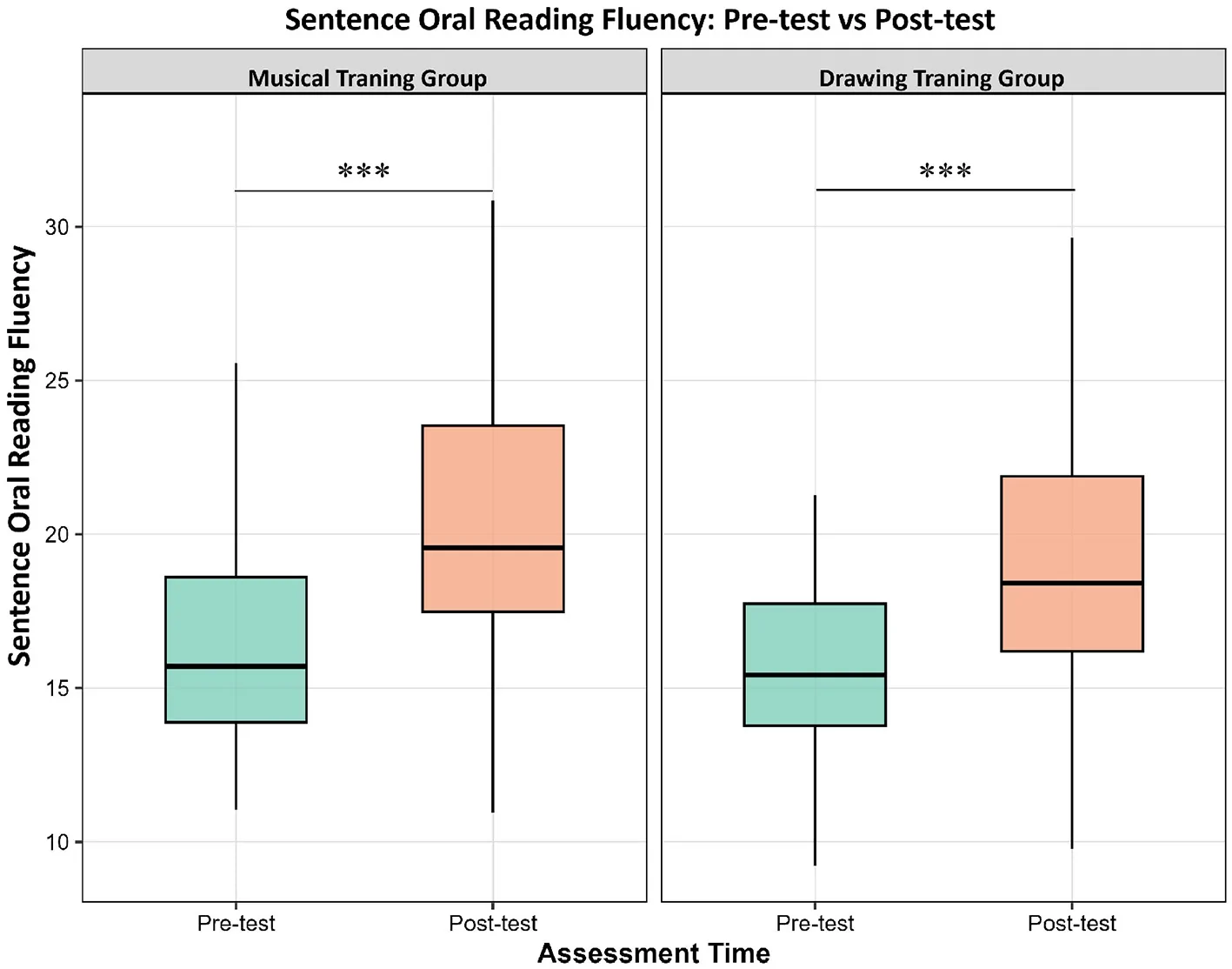
Sentence oral reading fluency scores from pretest to posttest by intervention group. The labels “Musical Training Group” and “Drawing Training Group” refer to the music intervention group and drawing intervention group, respectively. ***p < 0.001.

A 2 (assessment time: pretest, posttest) × 2 (intervention group: Musical Training Group, Drawing Training Group) repeated-measures ANOVA was conducted on the total sentence oral reading fluency score. The results showed a significant main effect of assessment time, F _(1, 58)_ = 8.735, *p* = 0.005, $\eta_{p}^{2}$ = 0.131. Simple effects analysis showed that sentence oral reading fluency scores were significantly higher at posttest (19.582 ± 0.605) than at pretest (16.982 ± 0.691). The main effect of intervention group was not significant, *F*_(1, 58)_ = 1.037, *p* = 0.313, $\eta_{p}^{2}$ = 0.018. The interaction between assessment time and intervention group was not significant, *F*_(1, 58)_ = 0.287, *p* = 0.594, $\eta_{p}^{2}$ = 0.005. See Figure 15 for details.

### 3.3 Discussion

Study 2 used a pretest–posttest intervention design to examine whether music-based rhythmic movement training could support children's working memory and reading ability. The results showed that children in the Musical Training Group had significantly higher musical ability scores at posttest than at pretest, suggesting that the training may have supported the development of musical ability. In the working memory task, children in the Musical Training Group also showed significantly higher accuracy at posttest than at pretest. For reading ability, the Musical Training Group showed significant pretest-to-posttest increases in antonym judgment and sentence proposition judgment scores, whereas no significant pretest-to-posttest changes were observed in the Drawing Training Group for these two tasks. Overall, these findings are broadly consistent with the results of Study 1. By using a longitudinal intervention design, Study 2 provides supplementary evidence for understanding the relationship among musical training, working memory, and reading ability in children.

## 4 General discussion

This study examined the relationship among musical training, working memory, and children's reading ability in children from multilingual backgrounds in frontier regions by combining a cross-sectional comparison with an intervention study. Study 1 showed that musically trained children performed better than non-musically trained children in musical ability, working memory, and some reading tasks, supporting H1a. Working memory mediated the relationship between musical ability and both antonym judgment and sentence proposition judgment, supporting H1b. Study 2 further implemented a 12 week music-based rhythmic movement training program consisting of 20 sessions among lower-grade children without prior musical training. The results showed that children in the music intervention group showed improvements in musical ability, working memory accuracy, antonym judgment, and sentence proposition judgment, supporting H2. Overall, the findings provide evidence from both an associational pathway and an intervention context, suggesting that working memory may represent a possible cognitive pathway linking musical training to the development of some reading-related abilities in children.

The findings of Study 1 and Study 2 together suggest a positive relationship between musical training and the development of some aspects of children's reading ability. This finding is consistent with previous studies showing associations between musical training and children's literacy skills (Gordon et al., 2015; Andrade et al., 2024). One possible explanation is that musical processing and reading processing share several basic cognitive resources. Previous studies have suggested that music and reading are not entirely independent processing systems but may overlap in phonological perception, rhythm processing, attentional control, working memory, and integration across sensory modalities (Moreno and Besson, 2006; Neves et al., 2022). These abilities are important foundations for reading development. Musical training may strengthen these underlying cognitive processes and thereby support reading processing in children (Sousa et al., 2022). From a neural perspective, this relationship may also be partly explained by functional changes in brain regions involved in language. For example, individuals with long-term musical training have been found to show stronger activation in Broca's area, a region involved in language processing, syntactic integration, and reading activities (Pantaleo et al., 2024). Therefore, neural plasticity induced by musical training may also extend to systems involved in language and reading, thereby supporting children's reading comprehension and language processing abilities (Neves et al., 2022; Pantaleo et al., 2024).

It is worth noting that the present study did not find a significant advantage of musical training in children's sentence oral reading fluency. Instead, sentence oral reading fluency showed a relatively stable grade-level advantage, suggesting that the relationship between musical training and children's reading ability may be task specific. The sentence oral reading fluency measure used in this study mainly reflected reading speed and accuracy within a limited time. Although this measure is suitable for assessing reading automaticity, it may not fully capture prosodic features in passage reading, such as rhythm, pausing, stress, and expressiveness (Kuhn and Stahl, 2003; Hsu et al., 2023). Previous studies have also suggested that reading fluency is not a single speed-based construct but includes multiple components, such as accuracy, automaticity, and prosodic expression (Miller and Schwanenflugel, 2008). Therefore, musical training may be more closely related to auditory rhythm processing, prosodic perception, and the organization of linguistic rhythm, whereas a sentence-level speed measure may not be sufficiently sensitive to capture its potential association with reading prosody or passage-level fluency (Zanto et al., 2024). In addition, oral reading fluency depends strongly on the automatization of word recognition, accumulated reading experience, and sustained reading practice (Lee and Yoon, 2017; Zimmermann et al., 2021). Compared with antonym judgment and sentence proposition judgment, sentence oral reading fluency is closer to a skilled reading output ability and may be more strongly influenced by grade level, reading experience, and school-based reading instruction. The stable grade-level effect observed in this study suggests that children's sentence oral reading fluency follows a clear developmental trend as age and learning experience increase. Thus, the additional contribution of short-term musical training may be relatively limited. For example, a training study with 6- to 8-year-old children found no significant advantage of the experimental group over the control group in overall literacy skills, although children with lower initial reading levels appeared to benefit more (Ahokas et al., 2025). Therefore, the effect of musical training on oral reading fluency may be moderated by children's initial reading level and developmental stage. For children who have already developed a certain level of reading automaticity, the effects of short-term training may be less likely to appear directly in a sentence-level reading speed measure.

The findings of Study 1 and Study 2 together suggest that working memory may represent an important cognitive mechanism linking musical training to the development of some reading-related abilities in children. This interpretation is consistent with previous research. A longitudinal study found a positive association between musical practice and the development of working memory in children and adolescents (Bergman Nutley et al., 2014). Intervention studies have also shown that musical training can benefit components of children's auditory working memory that involve executive control, particularly in tasks requiring processing and updating, such as backward span tasks (Nie et al., 2022). A recent meta-analysis further suggested that musical training is associated with benefits in early working memory, inhibitory control, and cognitive flexibility in children (Lu et al., 2025). Musical training may support the development of working memory because it relies heavily on auditory sequence processing, information maintenance, and cognitive control. During rhythm imitation, melody discrimination, singing, or instrumental learning, children need to maintain pitch, rhythm, and melodic sequences and adjust their responses according to external beats or feedback (Slevc et al., 2016; Miendlarzewska and Trost, 2014; Nie et al., 2022). These processes are closely aligned with the short-term maintenance, online processing, and continuous updating functions emphasized in working memory. They also involve the central executive system and cross-channel integration (Baddeley, 2000). In the present study, the mediating role of working memory was mainly observed in antonym judgment and sentence proposition judgment. Antonym judgment requires children to retrieve and maintain lexical meaning and to complete semantic comparison and matching. Sentence proposition judgment requires children to maintain word meanings, syntactic relations, and overall propositional information while judging whether the sentence meaning is valid. These processes require the temporary maintenance, updating, and integration of linguistic information within a short period and therefore depend strongly on working memory (Daneman and Merikle, 1996; Follmer, 2018). Thus, working memory may not directly enhance children's ability to read words aloud. Instead, it may support higher-level language comprehension processes, such as maintaining word meanings, comparing semantic information, integrating syntactic relations, and making propositional judgments. In this way, working memory may help explain the pathway through which musical training is related to the development of some reading-related abilities.

The results of Study 2 suggest that music-based rhythmic movement training may support the development of some reading-related abilities in daily educational practice. The music-based rhythmic movement training used in this study was developed for school-aged children in frontier regions of China. It drew on Dalcroze Eurhythmics and incorporated folk customs, nursery rhymes, musical instruments, and other culturally familiar materials from children's everyday lives, making it more suitable for children from multilingual backgrounds. The intervention results showed that, after participating in rhythmic movement training, children showed significant improvements in musical ability, working memory accuracy, antonym judgment, and sentence proposition judgment. These changes may be related to the integrated characteristics of rhythmic movement training. First, musical training usually requires children to continuously maintain and update rhythm, melody, and movement sequences. It therefore depends strongly on working memory storage, updating, and executive control (Miendlarzewska and Trost, 2014; Nie et al., 2022; Slevc et al., 2016). Repeated practice in such training contexts may also support the development of working memory (Bergman Nutley et al., 2014; Lu et al., 2025). Second, Dalcroze Eurhythmics emphasizes the body as the primary instrument for actively perceiving and expressing music. This view is consistent with embodied cognition, which highlights the interaction among the body, cognition, and the environment (Juntunen and Hyvönen, 2004). During training, children need to synchronize rhythm perception with bodily movement, thereby forming multisensory learning channels (Juntunen and Westerlund, 2001; Juntunen and Sutela, 2023). Third, music-based rhythmic movement training may involve the participation of broad neural networks (Herholz and Zatorre, 2012). Neuroimaging studies have reported that musical training is associated with gray matter volume in the prefrontal and occipital regions and with cortical thickness in the prefrontal and parietal regions (Sato et al., 2015; Liu et al., 2025). These regions are also important for higher-order cognitive processes, including working memory (Friedman and Robbins, 2022). Therefore, music-based rhythmic movement training may support children's reading-related abilities through working memory engagement, multisensory integration, and neural plasticity in related brain regions.

Although the present study found a positive relationship between musical training and the development of some reading-related abilities in children, and further highlighted the role of working memory in this relationship, several limitations should be acknowledged. Regarding measurement, this study used only a one-back letter task to assess working memory. This provides a relatively limited indicator of working memory. Future studies could include digit span, complex span, and higher-load n-back tasks to more comprehensively distinguish the roles of different components of working memory in the relationship between musical training and children's reading development. In addition, the assessment of reading fluency could be extended to include passage oral reading measures, which may improve sensitivity to the potential role of musical training in reading prosody and broader fluency. With respect to research design, Study 1 mainly examined overall differences between musically trained and non-musically trained children. Future research could further distinguish different forms of musical training, such as instrumental training, vocal training, and rhythmic movement training, and compare their associations with different components of working memory and reading ability. In Study 2, because of constraints in the real school setting, natural classroom-based cluster assignment was adopted within the school, and the intervention groups were allocated according to implementation feasibility. This approach may help reduce intervention contamination and improve ecological validity. However, future studies could use individual randomization, multilevel randomization at the class or school level, or crossover designs in larger samples and across multiple schools to improve internal validity and generalizability. Another limitation concerns the intervention duration and sample size. Study 2 did not examine the longitudinal mediation pathway through which musical training may influence reading ability, nor did it include long-term follow-up assessments. Future research could use larger samples, longer randomized controlled interventions, and delayed posttests to examine whether the effects of musical training are maintained over time. Such studies could also test whether changes in musical ability predict changes in reading ability through changes in working memory. Finally, the sample in this study was mainly drawn from frontier regions of Yunnan Province. The stability of the findings should therefore be further examined across different regions, language backgrounds, and educational resource conditions. At the same time, the music-based rhythmic movement training used in this study has practical value because it is low cost, easy to implement, accessible, and adaptable to local cultural contexts. It can be carried out in everyday educational settings, such as regular classrooms and school playgrounds, and can be combined with nursery rhymes, ethnic musical instruments, local festivals, and folk music activities. In future school practice, this type of training could be integrated into recess activities, club activities, or comprehensive practice courses. Schools could also make use of community and local cultural resources by inviting parents with musical expertise, local artists, or inheritors of intangible cultural heritage into the classroom. These practices may enrich both the training content and the forms of instruction, and may provide a more feasible pathway for supporting reading development among children from multilingual backgrounds in frontier regions and other educationally under-resourced areas.

## 5 Conclusion

This study examined the relationship among musical training, working memory, and reading ability in children from multilingual backgrounds in frontier regions. In Study 1, musically trained children performed better than non-musically trained children in musical ability, working memory, and some reading tasks. Working memory mediated the relationship between musical ability and specific reading outcomes, showing a partial mediating role in the relationship between musical ability and antonym judgment and a full mediating role in the relationship between musical ability and sentence proposition judgment. In Study 2, the music-based rhythmic movement training program, which lasted 12 weeks and consisted of 20 sessions, was associated with improvements in musical ability, working memory accuracy, antonym judgment, and sentence proposition judgment among children in the lower grades of primary school. Overall, these findings suggest that musical training may be positively related to abilities related to reading, and that working memory may serve as an important cognitive pathway in this relationship.

## Data Availability

The datasets for this article are not publicly available due to concerns regarding participant anonymity and privacy. Requests to access the datasets should be directed to the corresponding author.
