# Correction: The impact of musical training on reading ability in children: the mediating role of working memory

**DOI:** 10.3389/fpsyg.2026.1939872

**Published:** 2026-08-27

**Authors:** Chunjing Yang, Qijun Hu, Lin Fu, Yuanlong Jiang, Zhi Zhu, Han Ding, Wei Wang, Yun Tao, Xie Ma

**Affiliations:** 1 Faculty of Education, Yunnan Normal University, Kunming, China; 2 School of Psychology, Yunnan Normal University, Kunming, China

**Keywords:** musical training, music-based rhythmic movement training, reading ability, working memory, children from multilingual backgrounds

Affiliation “Faculty of Education, Yunnan Normal University, Kunming, China” was omitted from the paper. This affiliation has now been added for authors Chunjing Yang, Qijun Hu, Zhi Zhu, Yun Tao and Xie Ma.

Affiliation “School of Psychology, Yunnan Normal University, Kunming, China” was erroneously given as “Faculty of Psychology, Yunnan Normal University, Kunming, China”.

The original version of this article has been updated.

