# Supplementary material for: The impact of musical training on reading ability in children: the mediating role of working memory
Source: Front Psychol. 2026 Jul 7;17:1829093. doi: 10.3389/fpsyg.2026.1829093 (PMC13386267; doi:10.3389/fpsyg.2026.1829093)

Study 1 Parent/Guardian Written Informed Consent Form

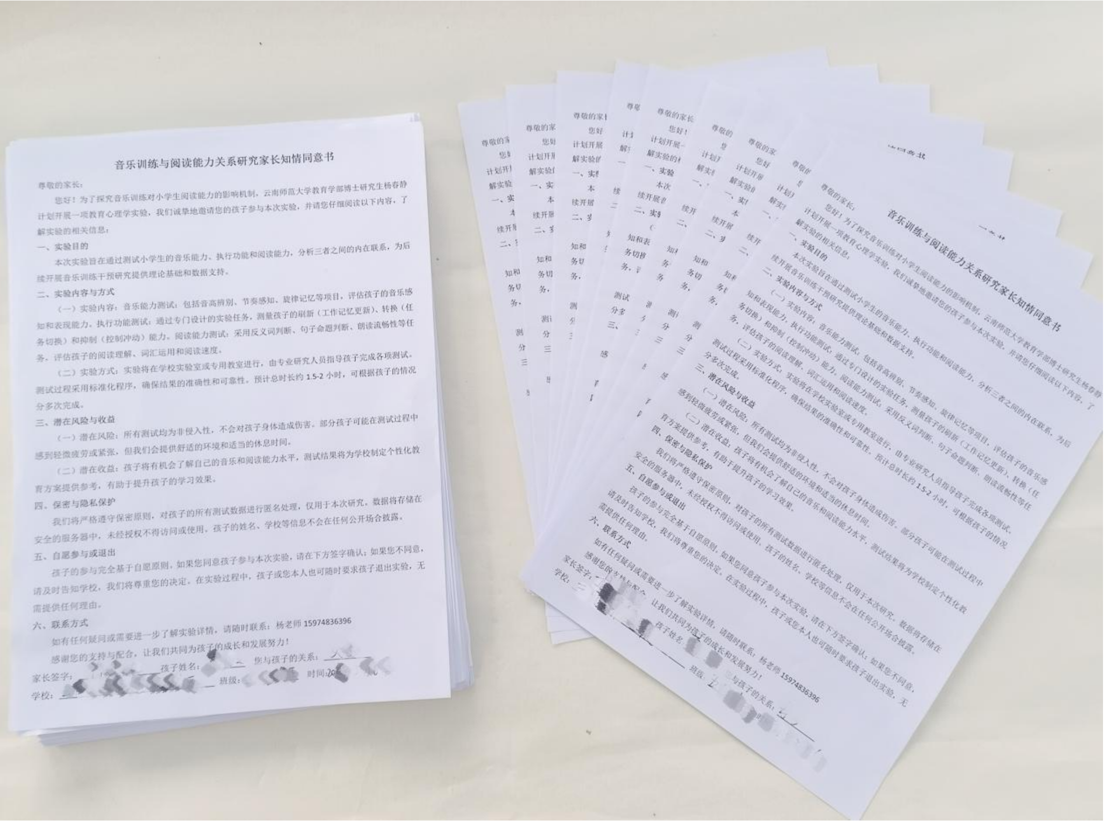

音乐训练与阅读能力关系研究家长知情同意书

尊敬的家长：

您好！为了探究音乐训练对小学生阅读能力的影响机制，云南师范大学教育学部博士研究生杨春静计划开展一项教育心理学实验，我们诚挚地邀请您的孩子参与本次实验，并请您仔细阅读以下内容，了解实验的相关信息：

一、实验目的

本次实验旨在通过测试小学生的音乐能力、执行功能和阅读能力，分析三者之间的内在联系，为后续开展音乐训练干预研究提供理论基础和数据支持。

二、实验内容与方式

（一）实验内容：音乐能力测试：包括音高辨别、节奏感知、旋律记忆等项目，评估孩子的音乐感知和表现能力。执行功能测试：通过专门设计的实验任务，测量孩子的刷新（工作记忆更新）、转换（任务切换）和抑制（控制冲动）能力。阅读能力测试：采用反义词判断、句子命题判断、朗读流畅性等任务，评估孩子的阅读理解、词汇运用和阅读速度。

（二）实验方式：实验将在学校实验室或专用教室进行，由专业研究人员指导孩子完成各项测试。测试过程采用标准化程序，确保结果的准确性和可靠性。预计总时长约 1.5-2 小时，可根据孩子的情况分多次完成。

三、潜在风险与收益

（一）潜在风险：所有测试均为非侵入性，不会对孩子身体造成伤害。部分孩子可能在测试过程中感到轻微疲劳或紧张，但我们会提供舒适的环境和适当的休息时间。

（二）潜在收益：孩子将有机会了解自己的音乐和阅读能力水平，测试结果将为学校制定个性化教育方案提供参考，有助于提升孩子的学习效果。

四、保密与隐私保护

我们将严格遵守保密原则，对孩子的所有测试数据进行匿名处理，仅用于本次研究。数据将存储在安全的服务器中，未经授权不得访问或使用。孩子的姓名、学校等信息不会在任何公开场合披露。

五、自愿参与或退出

孩子的参与完全基于自愿原则。如果您同意孩子参与本次实验，请在下方签字确认；如果您不同意，请及时告知学校，我们将尊重您的决定。在实验过程中，孩子或您本人也可随时要求孩子退出实验，无需提供任何理由。

六、联系方式

如有任何疑问或需要进一步了解实验详情，请随时联系：杨老师 15974836396

感谢您的支持与配合，让我们共同为孩子的成长和发展努力！

家长签字：______ 孩子姓名：______ 您与孩子的关系：______

学校：______ 班级：______ 时间：______

Study 1 Child Assent Form

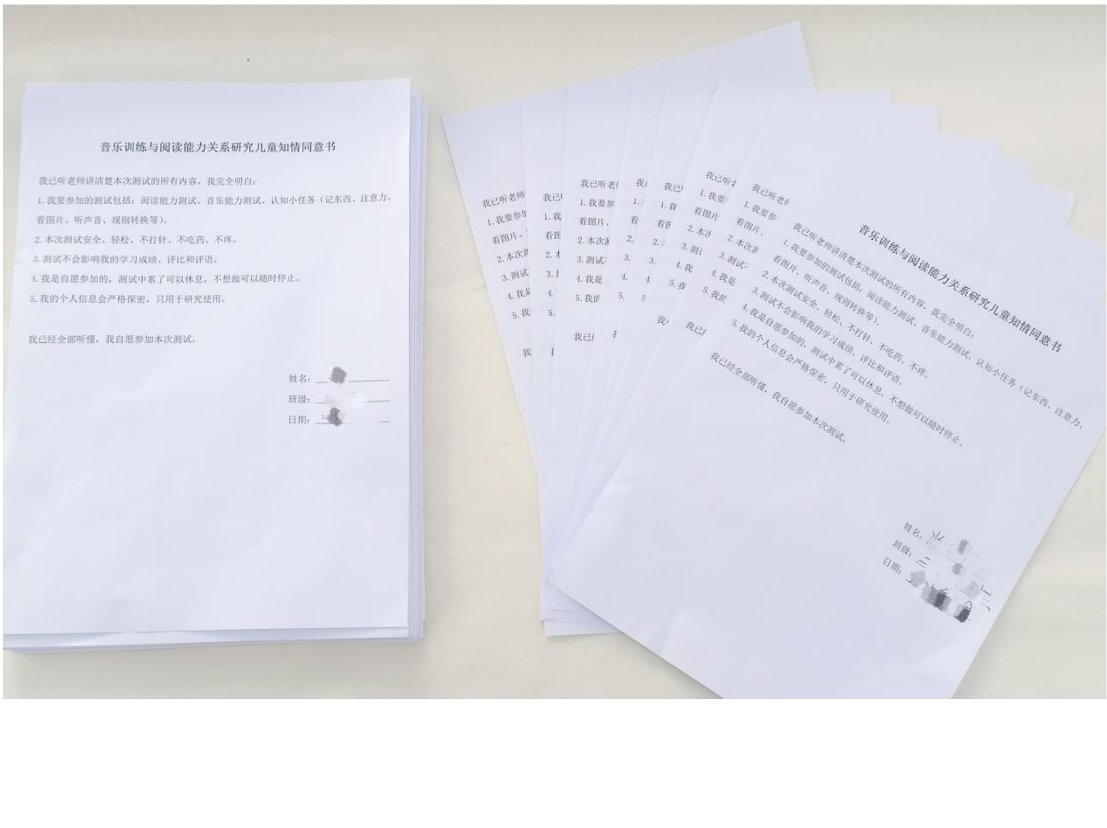

音乐训练与阅读能力关系研究儿童知情同意书

我已听老师讲清楚本次测试的所有内容，我完全明白：

1. 我要参加的测试包括：阅读能力测试、音乐能力测试、认知小任务（记东西、注意力、看图片、听声音、规则转换等）。
2. 本次测试安全、轻松、不打针、不吃药、不疼。
3. 测试不会影响我的学习成绩、评比和评语。
4. 我是自愿参加的，测试中累了可以休息，不想做可以随时停止。
5. 我的个人信息会严格保密，只用于研究使用。

我已经全部听懂，我自愿参加本次测试。

姓名：______

班级：______

日期：______

Study 2 Parent/Guardian Written Informed Consent Form

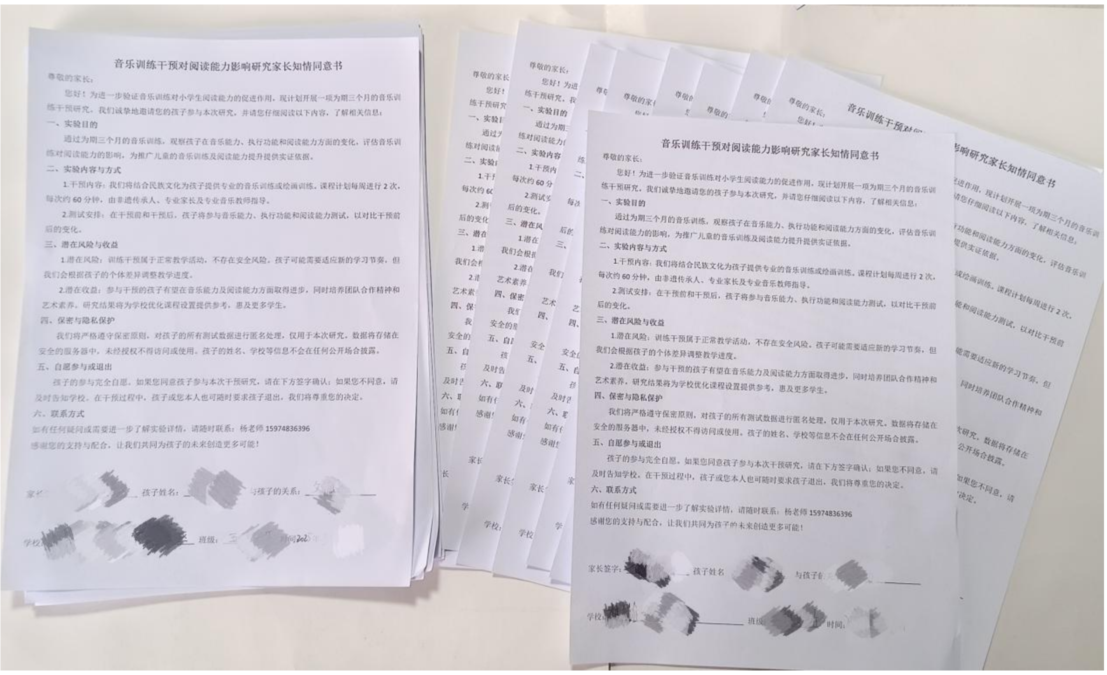

Study 2 Child Assent Form

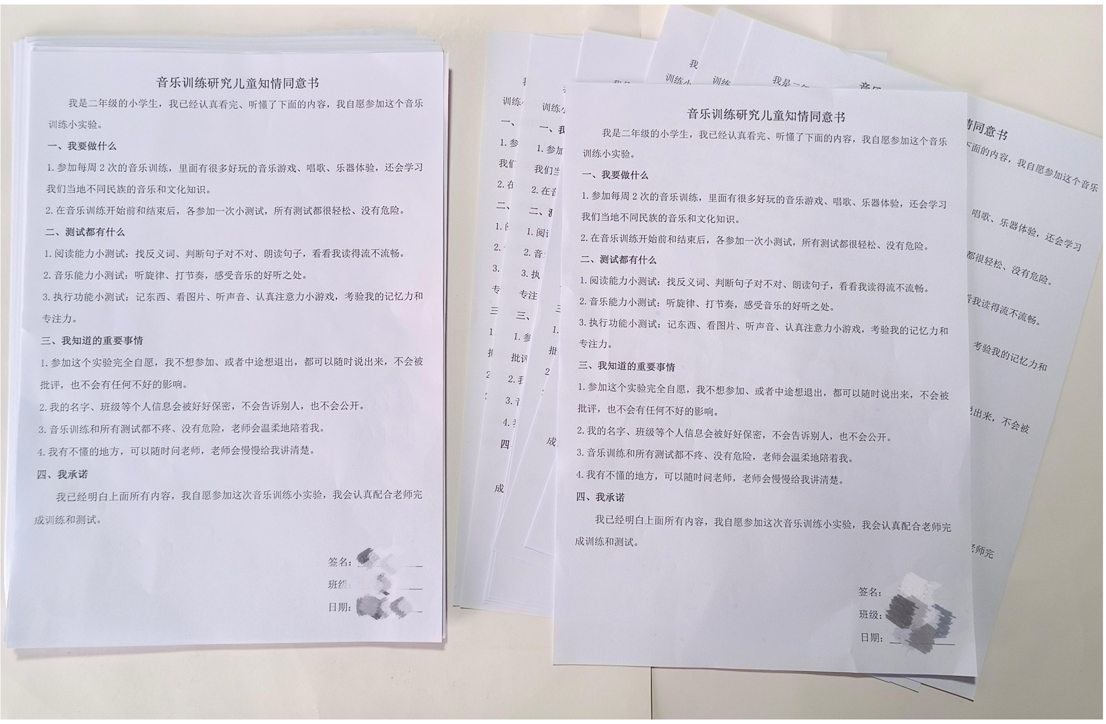

Supplement: Supplementary file 1 [file Supplementary_File_2.pdf]
